# Recovery of Arrested Replication Forks by Homologous Recombination Is Error-Prone

**DOI:** 10.1371/journal.pgen.1002976

**Published:** 2012-10-18

**Authors:** Ismail Iraqui, Yasmina Chekkal, Nada Jmari, Violena Pietrobon, Karine Fréon, Audrey Costes, Sarah A. E. Lambert

**Affiliations:** 1 Institut Curie, Centre de Recherche, Orsay, France; 2 CNRS, UMR3348, Centre Universitaire, Orsay, France; Duke University, United States of America

## Abstract

Homologous recombination is a universal mechanism that allows repair of DNA and provides support for DNA replication. Homologous recombination is therefore a major pathway that suppresses non-homology-mediated genome instability. Here, we report that recovery of impeded replication forks by homologous recombination is error-prone. Using a fork-arrest-based assay in fission yeast, we demonstrate that a single collapsed fork can cause mutations and large-scale genomic changes, including deletions and translocations. Fork-arrest-induced gross chromosomal rearrangements are mediated by inappropriate ectopic recombination events at the site of collapsed forks. Inverted repeats near the site of fork collapse stimulate large-scale genomic changes up to 1,500 times over spontaneous events. We also show that the high accuracy of DNA replication during S-phase is impaired by impediments to fork progression, since fork-arrest-induced mutation is due to erroneous DNA synthesis during recovery of replication forks. The mutations caused are small insertions/duplications between short tandem repeats (micro-homology) indicative of replication slippage. Our data establish that collapsed forks, but not stalled forks, recovered by homologous recombination are prone to replication slippage. The inaccuracy of DNA synthesis does not rely on PCNA ubiquitination or trans-lesion-synthesis DNA polymerases, and it is not counteracted by mismatch repair. We propose that deletions/insertions, mediated by micro-homology, leading to copy number variations during replication stress may arise by progression of error-prone replication forks restarted by homologous recombination.

## Author Summary

The appropriate transmission of genetic material during successive cell divisions requires the accurate duplication and segregation of parental DNA. The semi-conservative replication of chromosomes during S-phase is highly accurate and prevents accumulation of deleterious mutations. However, during each round of duplication, there are many impediments to the replication fork machinery that may hinder faithful chromosome duplication. Homologous recombination is a universal mechanism involved in the rescue of replication forks by rebuilding a replication apparatus at the fork (by mechanisms that are not yet understood). However, recombination can jeopardize genome stability because it allows genetic exchanges between homologous repeated sequences dispersed through the genome. In this study, we employ a fission yeast-based arrest of a single replication fork to investigate the consequences of replication fork arrest for genome stability. We report that a single blocked fork favours genomic deletions, translocations, and mutations; and this instability occurs during fork recovery by recombination. We also report that a single arrested fork that resumes its progression by recombination is prone to causing replication slippage mediated by micro-homology. We propose that deletions/duplications observed in human cancer cells suffering from replication stress can be viewed as scars left by error-prone replication forks restarted by recombination.

## Introduction

Maintenance of genome stability requires the faithful and accurate replication of the genetic material. Genome instability is a hallmark for most types of cancer and it is strongly associated with predisposition to cancer in many human syndromes (for a review, see [1], [2]). Genome instability is manifest at two levels: at the nucleotide level, resulting in base-substitutions, frame-shifts or in micro-insertions/deletions (referred to herein as mutations); and at the chromosomal level, resulting in duplications, deletions, inversions and translocations (referred to herein as gross chromosomal rearrangements or GCRs).

Genome instability during cancer development and in other human genomic disorders may be consequences of failures in chromosome replication (for a review, see [3], [4]). Origin spacing has recently been shown to cause chromosomal fragility at some human fragile sites [5], [6]. Impediments to replication fork movements *per se* may also cause genome instability [7]–[9]. Indeed, both slowing down and blockages to fork progression can lead to chromosomal fragilities or GCRs in human cells and yeast models [10]–[14]. However, how a blocked replication fork leads to genetic instability remains poorly understood.

In eukaryotes, DNA replication is initiated at numerous origins along linear chromosomes, and impediments to fork progression appear unavoidable during each S-phase (for a review, see [9], [15]). Impediments to fork progression can be caused by DNA lesions, by non-histone proteins tightly bound to DNA, by sequence-caused secondary structures such as cruciform structures and possibly G-quadruplexes, by nucleotide pool imbalance and by conflicts with transcription machinery (for a review, see [16], [17]). In case of failures in fork progression, DNA replication can be completed either by the recovery of the arrested fork by fork-restart mechanisms, or as a result of the progression of a converging fork which can be ensured by activation of dormant origins [7], [15], [18]. Fork restart is presumably essential in unidirectional replication regions, such as the rDNA locus, in regions of low densities of origins, such as some human fragile sites, and when two converging forks are both impeded [5], [19], [20].

To ensure faithful and complete DNA replication, cells coordinate DNA synthesis restart with specific pathways, including DNA replication checkpoint and homologous recombination mechanisms [17]. The integrity of replication forks is guaranteed by the DNA replication checkpoint that maintains the replisome in a replication-competent state to keep DNA polymerases at the site of nucleotide incorporation [21]. It remains unclear how the DNA replication checkpoint modulates replisome activities to maintain its function [21], [22]. The DNA replication checkpoint also regulates nuclease activities (*e.g.* Exo1 or Mus81) which contribute to preserving the integrity of stalled forks [23], [24]. If replisome function is lost or the replisome dissociates at broken replication forks, the resumption of DNA synthesis appears to require the replisome to be rebuilt. In *E.coli*, restart of a collapsed fork involves homologous recombination and the PriA helicase that allows replisome components to be loaded *de novo* on joint-molecule structures [25], [26]. In eukaryotes, the restart of collapsed or broken replication forks is dependent upon homologous recombination, but the mechanism of origin-independent loading of the replisome remains to be described [20], [27]–[30]. It has been proposed that the repair of a double-strand break (DSB) by recombination (break-induced replication, BIR) in budding yeast similarly involves the assembly of a replication fork (for a review, see [30]–[32]). When BIR occurs outside S-phase, recombination-dependent replication fork assembly can synthesise hundreds of kilobases (Kb). However, this DNA synthesis is highly inaccurate due to frequent template switching of nascent-strands and frame-shift mutations [33], [34].

We previously reported a system that displays replication fork arrest at a specific locus in the fission yeast *S. pombe*. The system is a polar replication fork barrier (RFB) regulated by the Rtf1 protein binding to its *RTS1* binding site [35]. The *RTS1*-RFB causes fork arrest because of a non-histone protein complex binding to the DNA. As proposed for other polar RFBs, the *RTS1*-RFB is thought to block fork progression by directly (contact between proteins and the replisome) or indirectly (topological constraint) affecting the replicative helicase activity and thereby preventing DNA unwinding [36], [37]. Recovery of the arrested fork occurs by a DSB-independent mechanism and involves the recruitment of recombination proteins at the *RTS1*-RFB site. We proposed that recombination proteins associate with unwound nascent strands that then anneal with the initial template to allow DNA synthesis to restart [11], [20]. The causative protein barrier then has to be removed either by DNA helicase or by the recombination machinery itself to allow fork-progression to resume [38]–[40]. Occasionally, the unwound nascent strand can mistakenly anneal with a homologous template in the vicinity of the collapsed fork, resulting in the restart of DNA synthesis on non-contiguous template. This incorrect template switch of nascent strands results in inversions and iso-acentric and dicentric chromosomes in ∼2–3% of cells/generation [11], [20]. Error-free template switching between sister-chromatids provides an efficient mechanism for filling-in single-stranded gaps left behind damage-induced stalled forks [41]. Inverted chromosome fusions in yeast and rare-genome rearrangements in human genomic disorders, may both be consequences of template switching between ectopic repeats associated with impeded replication forks [8], [14].

Here, we used the *RTS1*-RFB to investigate the consequences of fork collapse on genome instability. We report that recovery from a collapsed fork is associated with a high frequency of instability, with a single fork arrest increasing the rates of mutation, deletion and translocation by 10, 40 and 5 fold, respectively. We show that genetic instability associated with fork arrest is dependent on homologous recombination. Fork-arrest-induced GCRs (deletion and translocation) result from inappropriate ectopic recombination at the site of the collapsed fork. We also demonstrate that restoration of fork progression by homologous recombination results in error-prone DNA synthesis due to frequent replication slippage between short tandem repeats. We investigated the molecular mechanisms of this replication slippage and found that post-replication repair, including ubiquitination of PCNA or trans-lesions-synthesis (TLS) DNA polymerases, is not involved in fork-arrest-induced replication slippage. Micro-deletions/insertions flanked by micro-homology associated with copy number variations (CNVs) in cancer cells or in response to replication stress may therefore be scars left following the restoration of forks progression by homologous recombination.

## Results

### The conditional replication fork barrier *RTS1*

**Figure 1:**
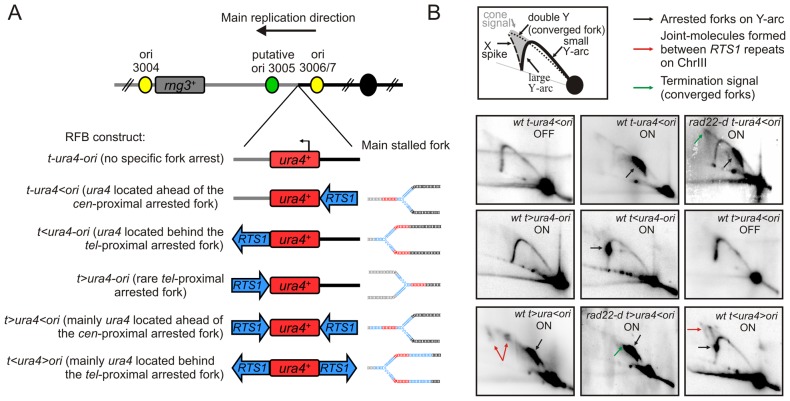
Conditional replication fork-arrest assays. A. Diagrams of fork-arrest constructs. *Centromere*-proximal and *telomere*-proximal regions are represented in black and grey, respectively. Strong or putative replication origins (ori) and the centromere are indicated by yellow, green and black circles, respectively. Blues arrows indicate the polarity of the *RTS1*-RFB. The *ura4^+^* gene is indicated in red and the arrow indicates its direction of transcription. Representations of the primary arrested fork structure are given for each construct. The name of each fork-arrest construct is given using the following nomenclature: “t” and “ori” refer to the telomere and the replication origin 3006/7, respectively; “<“ and ”>” indicate the *RTS1*-barrier and its polarity (< blocks replication forks moving from the ori3006/7 towards the telomere, and > blocks replication forks moving from the telomere towards the origin 3006/7. B. Diagrams of replication intermediates (RIs) within the A*se*I fragment analysed by 2DGE (top panel). Representative RIs analysed by 2DGE in indicated strains in OFF (Rtf1 being repressed) and ON (Rtf1 being expressed) conditions. Signal corresponding to arrested forks, joints-molecules (JMs) and termination structures are indicated by black, red and green arrows, respectively. Note that the *t>ura4-ori* construct does not result in a strong fork arrest as the *RTS1*-RFB is not orientated in the main direction of replication (see text for details).

We generated fork arrest constructs by manipulating the polar *RTS1*-RFB (Figure 1A). We introduced the *RTS1* sequence on the centromere-proximal (*cen*-proximal) side of the *ura4* locus, 5 kb away from the strong replication origin (ori) 3006/7 on chromosome III. This created the *t-ura4<ori* locus, in which “*t*” and “*ori*” refer to the telomere and the origin 3006/7, respectively; and “<” and“ >”refer to the *RTS1*-barrier and its polarity that is whether it blocks replication forks travelling from the ori 3006/7 towards the telomere or forks travelling from the telomere towards the ori 3006/7, respectively. We previously confirmed that forks moving from ori 3006/7 towards the telomere (*tel*) are efficiently blocked by the *RTS1*-RFB at the *t-ura4<ori* locus [35]. In this model system, fork arrest is activated by inducing the expression of *rtf1^+^* gene that is under control of the thiamine repressible promoter *nmt41*. Thus, the *RTS1*-RFB is inactivated by adding thiamine to the media and it is activated in thiamine-free media. Efficient induction of Rtf1 expression requires incubation for 12–16 hours in thiamine-free media. Replication intermediates were analysed by native 2-dimensional gel electrophoresis (2DGE). In conditions of Rtf1 expression, more than 95% of replication forks were blocked by the *RTS1-*RFB at the *t-ura4<ori* locus (see black arrow on Figure 1B, *t-ura4<ori* ON). Arrested forks were not detected without Rtf1 induction (Figure 1B, *t-ura4<ori* OFF) [20].

### Conditional fork-arrest constructs to investigate fork-arrest-induced genome instability

The *RTS1* sequence was inserted on the *tel*-proximal side of *ura4* creating the *t<ura4-ori* locus. 2DGE analysis of this construct revealed a strong fork arrest signal on the descending large Y arc (Figure 1A and 1B, *t<ura4-ori* ON). The *ura4^+^* gene, used in this system as a reporter to score genetic instability, is located behind the arrested fork when the *RTS1*-RFB is active at the *t<ura4-ori* locus and ahead of the arrested fork at the *t-ura4<ori* locus. This explains the distinct position of the arrested fork signal on the Y arc. Inversion of the *RTS1* sequence at the *tel*-proximal side of *ura4* created the *t>ura4-ori* locus and no fork arrest signal was detected for this construct by 2DGE when Rtf1 was expressed (Figure 1A and 1B, *t>ura4-ori* ON). Thus, *RTS1* behaves as a polar RFB at the *ura4* locus, and replication across this locus is strongly unidirectional due to the relative positions of the origins [42].

Introducing a second *RTS1* sequence, such that the two *RTS1* sequences are inverted repeats (IRs), created *t>ura4<ori* and *t<ura4>ori* loci (Figure 1A and 1B, *t>ura4<ori* and *t<ura4>ori* ON). Given the orientation of the polar *RTS1*-RFB in the *t<ura4>ori* strain, converging forks cannot be blocked. Whereas block of converging forks can virtually occur in the *t>ura4<ori* strain, 2DGE in this construct revealed that forks arrested on the *cen*-proximal side of *ura4* were efficiently recovered by recombination before forks are arrested on the *tel*-proximal side. Indeed, joint-molecules (JMs) resulting from recombination between *RTS1* repeats were detected by 2DGE (see red arrows on Figure 1B, *t>ura4<ori* and *t<ura4>ori* ON). Resolution of these JMs gives rise to chromosomal rearrangements [20]. In the absence of homologous recombination (*i.e.* in a *rad22-d* mutant), JMs were not detected and termination signals accumulated (see green arrow on Figure 1B, *t>ura4<ori rad22-d* strain). Similarly, termination signals accumulated in the *rad22-d t-ura4<ori* strain (see green arrow on Figure 1B, *t-ura4<ori rad22-d*), showing that, when arrested forks are not restarted by homologous recombination, the *RTS1*-RFB behaves as a hot spot for replication termination [20].

### A single fork arrest induces genomic deletions

**Figure 2:**
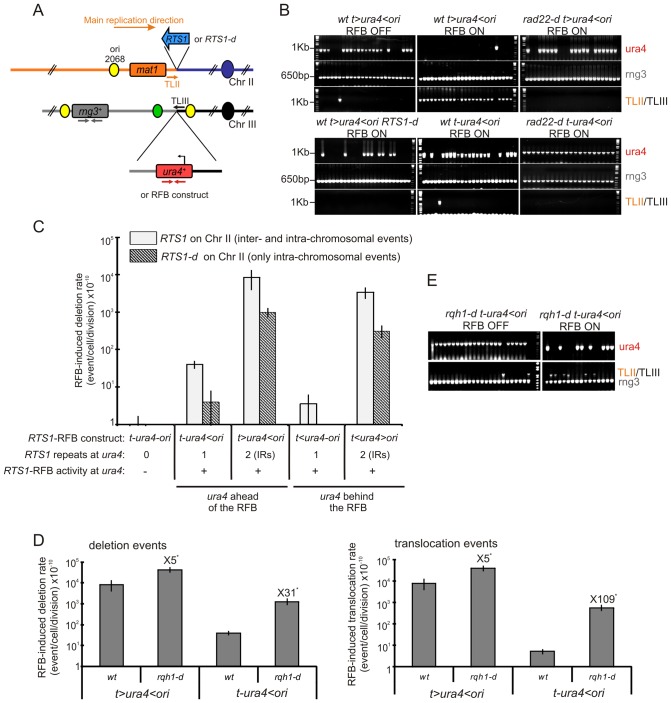
A single fork-arrest induces GCRs that are stimulated by inverted repeats near the site of fork arrest. A. Diagrams of chromosome II containing or not the *RTS1* sequence (blue arrow or *RTS1-d*) and of chromosome III containing *ura4^+^* alone or associated with *RTS1-*RFB constructs. The *RTS1* sequence maps near the *mat1* locus where it helps to ensure unidirectional replication [62]. Primers used for amplifying the 1 Kb *ura4* fragment or the 650 bp *rng3* fragment are depicted in red and grey, respectively. Primers used to amplify the translocation junction (1.2 kb) are represented in orange on chromosome II (TLII) and in black on chromosome III (TLIII). B. Representative PCR-amplifications from 5-FOA^R^ colonies of the indicated strains; ON and OFF refers to the *RTS1*-RFB being active or not, respectively. PCR products and their sizes are indicated on the figure. C. Effect of intra- and inter-chromosomal recombination between *RTS1* repeats on fork-arrest-induced genomic deletion. *RTS1*-RFB activity and *ura4* location with respect to the RFB are given for each construct. The % of deletion events, as determined by the PCR assay, was used to balance the rate of *ura4* loss. Then, the RFB-induced deletion rate was calculated by subtracting the rate obtained in the presence of thiamine (Rtf1 being repressed) from the rate obtained in the absence of thiamine (Rtf1 being expressed). The values reported are means of at least 3 independent median rates. Error bars correspond to the standard error (SE). D. Effect of Rqh1 on RFB-induced deletions (left) and translocations (right), as described for panel C. Error bars indicate SE. Statistically significant fold differences between the *rqh1-d* and the wild-type strains are indicated with an *. E. Representative PCR amplifications from 5-FOA^R^ colonies of the *rqh1-d t-ura4<ori* strain, as described for panel B. (Refer to Figure S1 for corresponding rates of deletion and translocation when Rtf1 is expressed or not).

We investigated fork-arrest-induced genome instability by selecting for cell resistance to 5-FOA^R^, the result of loss of *ura4^+^* function. Inducing fork-arrest at *t-ura4<ori* increased *ura4* loss 3 fold (Table 1). Rtf1 expression in the *t-ura4-ori* and *t>ura4-ori* strains did not cause site-specific fork-arrest at *ura4* as assessed by 2DGE and did not increase the rate of *ura4* loss. Thus, *ura4* loss results from the *RTS1*-RFB activity and not simply from the presence of *RTS1* and/or Rtf1 expression (Table 1). To investigate the nature of this genetic instability, primers were designed to amplify the *ura4* coding sequence and, as a control, the essential *rng3* gene, mapping 30 kb *tel*-proximal to *ura4*, that should not be rearranged (Figure 2A and 2B) [35]. The absence of *ura4* amplification was classified as a deletion event; sequencing of amplified *ura4* sequence was used to identify point mutation events (Figure 2B).

**Table 1:** Rates of *ura4* loss (including genomic deletion, translocation, and mutation events), calculated using the method of the median.

strains^a^	Rate of *ura4* loss^b^		Fold induction by Rtf1 expression (−/+ thiamine)^c^	Fold induction by the RFB (relative to the *t-ura4-ori* construct)
	+ thiamine (Rtf1 repressed)	− thiamine (Rtf1 expressed)		
*t-ura4-ori*	4.6±1	4.3±1.8	0.9	
*t-ura4<ori*	4.1±0.9	13.9±2.8	3.4 (p = 0.002)	3.2
*t-ura4<ori RTS1-d*	4.9±1.4	11.4±4.1	2.3 (p = 0.03)	2.6
*t<ura4-ori*	3.9±1.3	4.8±2.5	1.2	1.1
*t>ura4<ori*	5.8±2.1	97.4±51.5	16.8 (p = 0.004)	22.6
*t>ura4<ori RTS1-d*	4.9±2.2	19.5±3.3	4 (p = 0.006)	4.5
*t>ura4-ori*	3.2±1.7	3.6±1.8	1.1	0.8
*t<ura4>ori*	6.6±1.7	50.3±14.2	7.6 (p = 0.014)	11.7

a the following nomenclature is used: “t” and “ori” refer to the telomere and the replication origin 3006/7, respectively; “<“ and ”>” indicate the *RTS1*-RFB and its polarity (< blocks replication forks moving from the ori3006/7 towards the telomere, and > blocks replication forks moving from the telomere towards the origin 3006/7).

b event/cell/division ×10^−8^ ± standard error. Values are means of at least 3 independent rates.

c statistical significance was determined using the nonparametric Mann-Whitney U test.

A single arrested fork at the *t-ura4<ori* locus was sufficient to increase the rate of genomic deletion up to 40 times over spontaneous events (*i.e.* in the *t-ura4-ori* strain, p = 0.006) (Figure 2C and Figure S1A). Fork-arrest-induced deletion was recombination-dependent. Spontaneously (*i.e.* when the *RTS1*-barrier was inactivated), the rate of genomic deletion in *rad22-d* or *rhp51-d* strains was higher than that in the wild-type strain (Figure S1B). Nonetheless, no further increase in the rate of genomic deletion was observed in the surviving *rad22-d* or *rhp51-d* cells upon activation of the *RTS1*-barrier (Figure S1B, *t-ura4<ori*). Frequent spontaneous genomic deletion in the *rad22-d* or *rhp51-d* strains is consistent with previous reports showing that mutations in recombination genes are associated with an increase level of GCRs [14], [43], [44]. Deleting the natural *RTS1* sequence from chromosome II abolished deletion events at collapsed forks, indicating that fork-arrest-induced deletion was also mediated by inter-chromosomal recombination (Figure 2C and *t-ura4<ori RTS1-d* on Figure S1A). Thus, these data are consistent with the view that homologous recombination makes a major contribution to suppressing genome instability, but can occasionally drive non allelic recombination events leading to GCRs [35], [45].

We detected no fork-arrest-induced deletion in the *t<ura4-ori* strain, in contrast to the *t-ura4<ori* strain (Figure S1A and Figure 2C). The *ura4* marker is located behind and ahead of collapsed forks in the *t<ura4-ori* and *t-ura4<ori* strains, respectively (Figure 1A). Therefore, replicated regions, located behind collapsed forks, do not display instability, and fork-arrest-induced deletion occurs within unreplicated regions immediately in front of arrested forks. Overall, our data establish that genomic deletion at collapsed forks results from inappropriate recombination between ectopic sequences during the process of fork recovery by recombination proteins.

### Inverted repeats stimulate fork-arrest-induced deletion by promoting inter- and intra-chromosomal recombination

Inverted repeats (IRs) are structural elements often associated with genome rearrangements [11], [14], [46], [47]. We investigated the effects of IRs in the vicinity of the *RTS1*-RFB on fork-arrest-induced genomic deletion. We first compared the *t>ura4<ori* strain (IRs flanking *ura4*) to the *t-ura4<ori* strain (no IRs near the *RTS1*-RFB). The rate of fork-arrest-induced genomic deletion was 200 times higher in the *t>ura4<ori* than that in the *t-ura4<ori* strain (p = 0.009, Figure 2C and Figure S1A). Thus, intra-chromosomal ectopic recombination permitted by the *RTS1* sequence on the *tel*-proximal side of *ura4* accounted for 99.5% of the genomic deletions observed in the *t>ura4<ori* strain (Figure 2C, compare with *t-ura4>ori*). Preventing inter-chromosomal recombination by deleting *RTS1* from the chromosome II (*t>ura4<ori RTS1-d*) abolished 90% of deletion events (Figure 2C and Figure S1A). Thus, genomic deletions induced by fork-arrest near IRs are due to inter- and intra-chromosomal recombination events. In support of this, stimulation of fork-arrest-induced deletion by IRs is mediated by homologous recombination. Indeed, the rate of genomic deletion was not increased upon induction of the *RTS1-*RFB in the surviving population of *t>ura4<ori rad22-d* and *rhp51-d* strains (Figure S1B). These data indicate that IRs favour genomic deletion at collapsed forks by promoting inappropriate inter- and intra-chromosomal recombination during fork recovery by recombination proteins.

We verified that our data were not influenced by the orientation of IRs or by rare blocking of converging forks in the *t>ura4<ori* strain. We analysed the *t<ura4>ori* construct in which *RTS1* repeats are in the opposite orientations relative to the *t>ura4<ori* construct, such that forks converging towards *ura4* cannot be blocked (Figure 1). The rate of fork-arrest-induced genomic deletion was 1,000 times higher in the *t<ura4>ori* than that in the *t<ura4-ori* strain, that does not contain IRs near the *RTS1*-RFB (p = 0.008, Figure 2C and Figure S1A). Thus, intra-chromosomal recombination, permitted by the *RTS1*-RFB sequence on the *cen*-proximal side of *ura4*, accounted for nearly 100% of the genomic deletions observed in the *t<ura4>ori* strain (Figure 2C, compare with *t<ura4-ori*). Preventing inter-chromosomal recombination by deleting *RTS1* from the chromosome II (*t<ura4>ori RTS1-d*) abolished 90% of deletion events (Figure 2C and Figure S1A). Importantly, the deletion rates for the *t<ura4>ori* and *t>ura4<ori* strains were not significantly different (Figure 2C), showing that IRs cause genomic deletion at collapsed forks irrespective of their orientations and independently of blockage of converging forks.

### A single collapsed fork induces translocations that are stimulated by IRs

Fork-arrest at *t>ura4<ori* results in translocations between ectopic *RTS1* repeats on chromosomes II and III [35]. We investigated the influence of IRs on fork-arrest-induced translocation. We designed primers to amplify the predicted translocation junction between chromosomes II and III (TLII and TLIII on Figure 2A and 2B). A single arrested fork at the *t-ura4<ori* locus was sufficient to increase the translocation rate to 5 times higher than the spontaneous rate (p = 0.002, Figure 2D and Figure S1C). The translocation rate for the *t>ura4<ori* construct (containing IRs) was 1,500 fold higher than that for the *t-ura4<ori* strain that does not contain IRs near the *RTS1*-RFB (p = 0.009, Figure 2D and Figure S1C). Thus, intra-chromosomal recombination accounted for nearly 99% of translocation events observed in the *t>ura4<ori* construct (Figure 2D and Figure S1C, compare with *t-ura4<ori*). No translocation events were detected when inter-chromosomal recombination was prevented by deleting *RTS1* from the chromosome II (*t>ura4<ori RTS1-d* on Figure 2B). Therefore, as reported for genomic deletions, fork-arrest-induced translocation associated with IRs is due to inter- and intra-chromosomal ectopic recombination. No translocations were detected in the *t<ura4>ori* strain (data not shown), so we cannot formally exclude the possibility that fork-arrest-induced translocations in the *t>ura4<ori* strain was caused by blocking of converging forks. However, as no translocation event occurred in the absence of Rad22^Rad52^ or Rhp51^Rad51^, it is most likely that translocations occur during fork recovery by recombination (Figure 2B and [35]). Overall, our data indicate that recovery of a single collapsed fork causes translocations and IRs near the site of fork-arrest stimulate translocations by promoting inappropriate inter- and intra-chromosomal recombination.

Fork-arrest-induced GCRs are caused by inter- and intra-chromosomal recombination. We noticed a slightly greater contribution of intra- than inter-chromosomal recombination (Figure 2C). This is consistent with ectopic recombination preferentially occurring at the most proximal homologous sequence, as previously reported [48]. Nonetheless, the rate of fork-arrest-induced deletion in the *t>ura4<ori* strain (8.4 10^−7^) was not simply the sum of the rates of intra-chromosomal recombination events (9.9 10^−8^ in the *t>ura4<ori RTS1-d* strain) and inter-chromosomal recombination events (4 10^−9^ in the *t-ura4>ori* strain). Similar reasoning can be applied for the *t<ura4>ori* strain. Thus, independent intra- and inter-recombination events cannot themselves explain high rate of GCRs induced by fork arrest near IRs. Therefore, we infer that there is interplay between inter- and intra-chromosomal recombination such that fork-arrest-induced GCRs may involve recombination between three homologous sequences (tri-parental recombination).

### The RecQ helicase Rqh1 prevents GCRs at collapsed forks

To confirm that fork-arrest-induced GCRs are the result of inappropriate ectopic recombination during fork recovery, we analysed the involvement of the RecQ helicase Rqh1. We previously reported that Rqh1 limits inappropriate template switching of stalled nascent strands without affecting the efficiency of fork restart [20]. In the *t-ura4<ori* construct (in which only inter-chromosomal recombination is possible), fork-arrest-induced deletion and translocation rates were 31 and 109 times higher in the *rqh1-d* strain than that in the wild-type control, respectively (p = 0.0003, Figure 2D–2E and Figure S1C). For the *t>ura4<ori* construct (containing IRs near fork-arrest), fork-arrest-induced deletion and translocation rates were 5 times higher in the *rqh1-d* than that in the wild-type control (p = 0.0007, Figure 2D–2E and Figure S1C). Thus, Rqh1 limits GCRs at collapsed forks by preventing inappropriate ectopic recombination during the process of fork recovery by recombination proteins.

### A single fork arrest induces mutations

**Figure 3:**
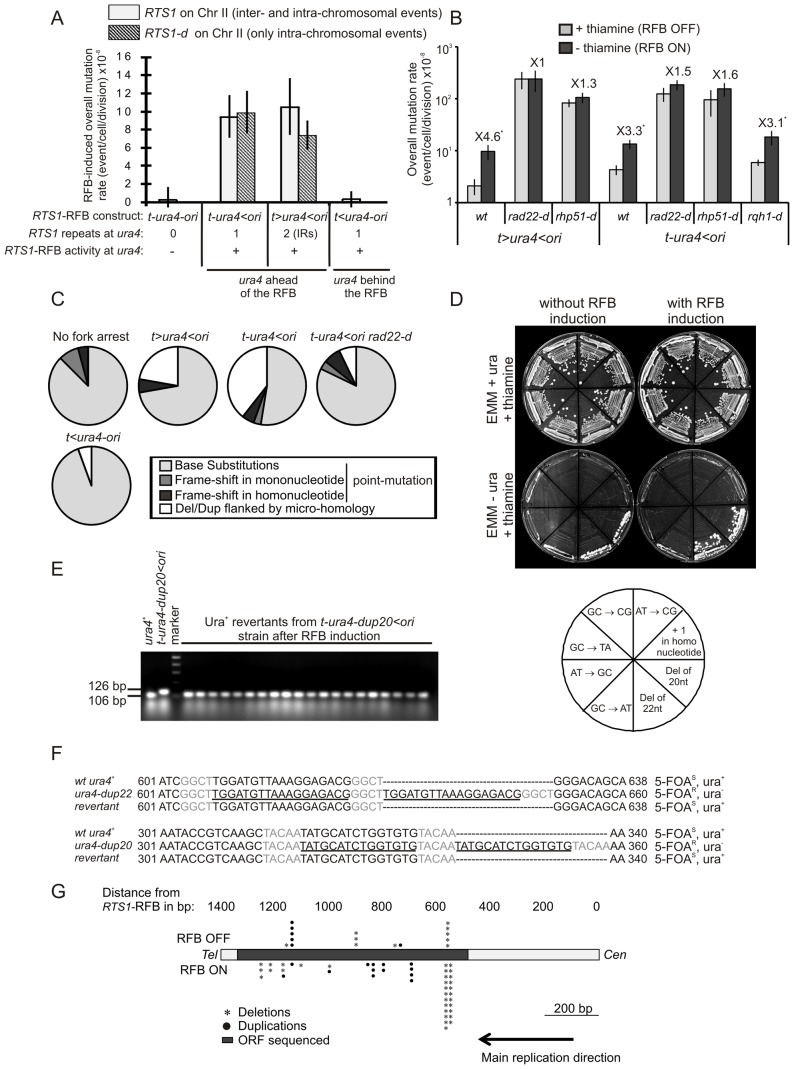
Fork-arrest induces mutations. A. Effect of intra- and inter-chromosomal recombination between *RTS1* repeats on fork-arrest-induced mutation rates (base-substitutions, frame-shifts and small insertions or deletions between short tandem repeats). *RTS1*-RFB activity and *ura4* location with respect to the RFB are given for each construct. The % of mutation events, as determined by the PCR assay and sequencing, was used to balance the rate of *ura4* loss. Then, the RFB-induced mutation rate was calculated by subtracting the rate obtained in the presence of thiamine (Rtf1 being repressed) from the rate obtained in the absence of thiamine (Rtf1 being expressed). The values reported are means of at least 3 independent median rates. Error bars correspond to SE. (Refer to Figure S2 for corresponding rates of mutation when Rtf1 is expressed or not). B. Rate of mutation for indicated strains; ON and OFF refers to the *RTS1*-RFB being active or not, respectively. The % of mutation events, as determined by the PCR assay and sequencing, was used to balance the rate of *ura4* loss. The values reported are means of at least 3 independent median rates. Error bars correspond to SE. Statistically significant fold differences in mutation rates between the “OFF” and “ON” conditions are indicated with an *. C. Spectra of mutation events in indicated strains upon RFB induction (refer to Table 2 for exact numbers and to Figure S3 for mapping of deletions/duplications and their features). D. Strains harbouring the *ura4* alleles with a single base-substitution or frame-shift or duplication of 20 or 22 nt, together with the *RTS1*-RFB in the *t-ura4<ori* configuration were streaked onto the indicated media after cell growth with (RFB “OFF”) or without (RFB “ON”) thiamine. The bottom diagram indicates strain positions and the mutation events required to obtain Ura^+^ revertants. E. PCR analysis of Ura^+^ revertants isolated from the *t-ura4-dup20<ori* strain (duplication of 20 nt in *ura4*) after RFB induction. With the primers used, a 106 bp fragment is amplified from the *ura4^+^* strain and a 126 bp fragment is amplified from the *t-ura4-dup20<ori* strain. F. Sequence alignments of *ura4-dup22*, *ura4-dup20*, *ura4^+^* alleles and corresponding Ura^+^ revertants. Micro-homologies are indicated in grey and duplicated sequences are underlined in black. The phenotype of each allele is indicated on the figure. G. Map of deletion and duplication events within the *ura4* ORF.

We analysed the effects of collapsed forks on the mutation rate. We sequenced the *ura4* coding sequence from 5-FOA^R^ isolated cells and identified base-substitutions, frame-shifts and small insertions and duplications between short tandem repeats (Table 2). A single collapsed fork in the *t-ura4<ori* strain increased the overall mutation rate up to 10 times over spontaneous events (Figure 3A, p = 0.003). Similar increases in the overall mutation rate were found for the strains with IRs near the arrested fork and those with *RTS1* deleted from chromosome II (Figure 3A and Figure S2A). Thus, fork-arrest-induced mutation is not mediated by inappropriate ectopic recombination. Induction of the *RTS1*-RFB in the *t<ura4-ori* strain did not increase the mutation rate of the *ura4* gene. Thus, as for GCRs, replicated regions behind arrested forks are not prone to mutation. This observation rules out the hypothesis that fork-arrest-induced mutation is a consequence of the accumulation of damaged single-stranded DNA behind collapsed forks (see discussion). Our data suggest that recovery from collapsed forks results in error-prone DNA-synthesis.

**Table 2:** Mutations spectra in the indicated strains.

	*t-ura4-ori* a		*t>ura4-ori*		*t-ura4<ori*		*t>ura4<ori*		*t<ura4-ori*		*t-ura4<ori rad22-d*	
Rtf1 expression	−	+	−	+	−	+	−	+	−	+	−	+
Fork arrest at *ura4*	−	−	−	−	−	+	−	+	−	+	−	+
Transitions^b^												
GC→AT	5			1	7	14	6	2		5	5	5
AT→GC	3		1	2	4	9	1	4	4	7	4	6
Transversions^b^												
GC→TA	3	5	1		45	20	5	2	4	2	3	4
GC→CG	1	3	4	2	2	3	2			1	8	5
AT→CG	3	3	1	1	6	4	2	2		1	4	
AT→TA		1	5	3	4	2	4	3	3	1	2	3
Rate of base substitutions^c^	4.6±1.1	3.7±1.5	3.3±1.6	4.2±1.3	3.4±0.9	6.4±1.2^d^	2.6±1.1	6.5±1.5^d^	2.5±1.3	3.3±1.7	115±34	152±33
+ Frame-shift^b^:												
in homo-nt		1							1			
in mono-nt		1	2	1	2	1	1		1		1	
- Frame-shift^b^:												
in homo-nt					1	4		1			1	2
in mono-nt					1	3			1			1
Rate of frame-shift^c^		0.6±0.25	0.5±0.3	0.5±0.1	0.2±0.06	1±0.2^d^	0.1±0.06	0.5±0.1	0.7±0.4		8.7±2.6	20.4±4.4
Del/Dupl^b^:												
Deletions					9	27		4		1		2
Duplications					5	12						
Rate of Del/Dupl^c^		<0.3		<0.5	0.7±0.2	4.9±1	<0.1	2.1±0.5	<0.2	0.2±0.1	<3.7	13±2.8
Other (complexe)					1							
Total	15	14	14	10	87	99	21	18	14	18	28	28

a the following nomenclature is used: “t” and “ori” refer to the telomere and the replication origin 3006/7, respectively; “<“ and ”>” indicate the *RTS1*-RFB and its polarity (< blocks replication forks moving from the ori3006/7 towards the telomere, and > blocks replication forks moving from the telomere towards the origin 3006/7).

b number of events.

c event/cell/division ×10^−8^ ± standard error. The % of each mutation events was used to balance the rate of mutation.

d value not significantly different from those for the *t-ura4-ori* strain (spontaneous events).

### Collapsed forks result specifically in replication slippage

We then analysed the spectra of mutations found in the *ura4* ORF by sequencing the PCR products. The rates of base-substitutions and frame-shifts were not significantly increased by the *RTS1*-RFB activity over spontaneous events (*i.e.* compare to *t-ura4-ori* strain, Figure 3C and Table 2). In contrast, the rate of deletions and duplications (Del/Dup) flanked by short homology was increased by 7 times over spontaneous events in the *t-ura4<ori* strain, but not in the *t<ura4-ori* strain (Figure 3C and Table 1). These data further confirm that fork-arrest does not promote mutation events behind collapsed forks.

We used reversed mutation assays to test if fork-arrest at the *RTS1*-RFB specifically induced Del/Dup mutations. We made use of strains harbouring a single mutation within the *ura4* ORF: either a single base-substitution or a −1 frame-shift in homo-nucleotide (Figure S2B). We also studied strains harbouring either a duplication of 20 or 22 nt flanked by 5 or 4 bp of micro-homology, respectively (defined as *ura4-dup20* and *ura4-dup22*, Figure S2B). These non-functional *ura4^−^* alleles were inserted in front of the *RTS1*-RFB in the *t-ura4<ori* configuration and we then tested whether fork arrest could restore a functional *ura4* ^+^ gene. Activation of the *RTS1*-RFB at *ura4* increased the frequency of Ura^+^ revertants up to 15 and 7 times in strains harbouring *ura4-dup22* and *ura4-dup20*, respectively (Figure 3D and Figure S2B). Thirty Ura^+^ colonies were studied by PCR and all gave a product of the same size as the wild-type *ura4^+^* gene: they had therefore lost the duplication (Figure 3E and data not shown). Sequencing the full *ura4* ORF confirmed that Ura^+^ revertants contained an intact *ura4^+^* sequence, showing that the reversion of these alleles was due solely to the precise deletion of 20 or 22 nt (Figure 3F and data not shown). In contrast, activation of the *RTS1*-RFB did not increase the frequency of Ura^+^ revertants of strains harbouring *ura4* alleles with a single base-substitution or a −1 frame-shift (Figure 3D and Figure S2B). Thus, collapsed forks tend to induce deletion events between short tandem repeats rather than base-substitution or frame-shift mutations.

Among Del/Dup events, deletions represented the two-third of events in the *t-ura4<ori* strain (Table 2). The median size of Del/Dup events was 24 and 22 nt respectively, and Del/Dup occurred between short direct repeats 1 to 10 nt long (Figure S3). Thus, the *ura4-dup20* and *ura4-dup22* alleles used in the reverse mutation assay were representative of the Del/Dup events observed. Del/Dup flanked by micro-homology result from intra-molecular template switching mechanisms in which nascent strands dissociate from the template and misalign with the template when restarting the elongation step. This leads to loop formation, either in the nascent strand or in the template, resulting in duplication or deletion events, respectively [49]. Consequently, we will hereafter refer to Del/Dup as replication slippage. Replication slippage was observed all along the *ura4* ORF and up to 1.2 kb ahead of the arrested fork, even if a hot spot of deletion was present 500 bp away from the *RTS1-*RFB (Figure 3G and Figure S3B). Thus, our data suggest that the DNA synthesis is prone to replication slippage at least for the first 1,200 nt synthetized during the recovery of collapsed forks. Inaccuracy of DNA synthesis on further distances was not directly addressed.

### Replication slippage results from error-prone DNA synthesis during fork recovery

**Figure 4:**
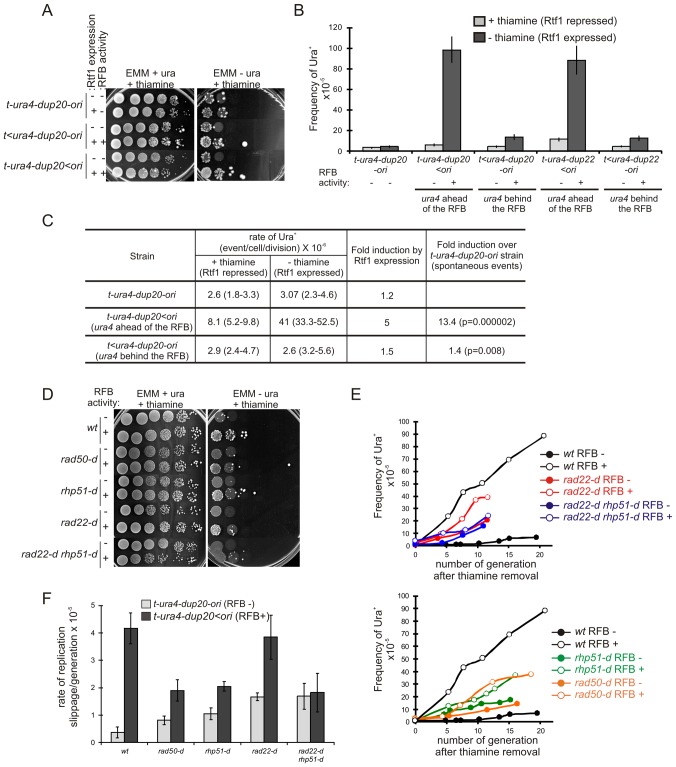
Fork recovery by homologous recombination results in replication slippage. A. Serial tenfold-dilutions from indicated strains (*t-ura4-dup20-ori* associated or not with the *RTS1*-RFB) were spotted onto the indicated media after cell growth with (Rtf1 -, repressed) or without (Rtf1 +, expressed) thiamine. *RTS1*-RFB activity is given for each construct and condition. B. Frequency of Ura^+^ revertants in indicated strains after cell growth with (Rtf1 repressed) or without (Rtf1 expressed) thiamine. The *RTS1*-RFB activity is given for each construct and condition. Values correspond to the mean of at least three independent experiments and error bars correspond to the standard error of the mean (SEM). C. Rate of replication slippage in the indicated strains and conditions. The rate of Ura^+^ revertants was calculated using the method of the median from at least 11 independent cultures. Values in brackets indicate the 95% confidence interval. Statistical significance was detected using the nonparametric Mann-Whitney U test. D. Serial tenfold-dilutions from the strains indicated spotted onto the media indicated after cell growth without thiamine. *RTS1*-RFB activity “–” refers to the strain *t-ura4-dup20-ori* and “+” refers to the strain *t-ura4-dup20<ori*. E. Kinetics of Ura^+^ revertants frequency for the strains indicated as a function of the number of generations after thiamine removal. *RTS1*-RFB activity “–” refers to the strain *t-ura4-dup20-ori* and “+” refers to the strain *t-ura4-dup20<ori*. The values reported are the means of two experiments. F. The rate of replication slippage/generation for the strains indicated with (*t-ura4-dup20<ori*) or without (*t-ura4-dup20-ori*) the active *RTS1*-RFB. The rate was calculated from the slope of the curves presented in panel F. The values reported are means of three independent experiments and error bars correspond to SE.

To confirm that replication slippage occurs as forks recover, and not behind the fork in the DNA already replicated, we inserted the *ura4-dup20* or the *ura4-dup22* allele either behind (in the *t<ura4-ori* configuration) or in front of the *RTS1*-RFB (in the *t-ura4<ori* configuration) (Figure 4). This allows the analysis of the same event of replication slippage behind and ahead of collapsed forks. In the *t-ura4<ori* configuration, induction of the *RTS1-*RFB resulted in a 8 and 16 fold increases in the replication slippage frequency for the *ura4-dup20* and *ura4-dup22* alleles, respectively (Figure 4A and 4B). Similar increases in the rate of replication slippages were observed (Figure 4C). In contrast, in the *t<ura4-ori* background, the frequency of replication slippage was induced by only 2–3 fold by the *RTS1-*RFB (Figure 4B–4C). These data confirm that DNA located ahead of collapsed forks is more prone to replication slippage than replicated DNA adjacent to arrested forks, further evidence that replication slippage arises during fork recovery.

### Replication slippage results from forks restarted by recombination

Replication slippage occurs in DNA in front of (and not behind) the arrested fork, this DNA being replicated only after restart of the fork. Thus, a defect preventing fork recovery would be expected to abolish the error-prone DNA synthesis during restart. We analyzed fork-arrest-induced mutation in recombination mutants in which collapsed forks at the *RTS1*-RFB cannot recover, resulting in cell death. Induction of the *RTS1*-RFB did not increase the overall mutation rate in the surviving populations of *t>ura4<ori* or *t-ura4<ori rad22-d* and *rhp51-d* strains (Figure 3B). In addition, only 7% of mutation events in the survivors of the *rad22-d t-ura4<ori* strain were Del/Dup mutations, compared to 40% in the wild-type strain (Figure 3C and Table 1). We currently cannot assess mutation events associated with defects in fork recovery because this appears to be lethal in the absence of recombination. Nevertheless, our data are consistent with fork-arrest-induced replication slippage being dependent on homologous recombination. The *rad22-d* and *rhp51-d* strains are themselves spontaneously mutagenic. Consequently, any small increase in the fork-arrest-induced mutation rate might be masked by the high frequency of spontaneous 5-FOA^R^ cells in *rad22-d* and *rhp51-d* strains. We therefore used a more specific mutation assay, based on the *ura4-dup20* allele, to determine the rate of replication slippage induced by the *RTS1*-RFB over spontaneous events.

Strains carrying mutations in recombination genes grow slowly, so replication slippage was scored as a function of the number of generations following thiamine removal (*i.e.* generations subject to fork arrest at *ura4*) (Figure 4D and 4E). In the wild-type strain, fork arrest at the *RTS1*-RFB resulted in a 10 fold-increase in the frequency of replication slippage, as expected. In recombination mutants (*rad50-d*, *rhp51-d* and *rad22-d*), fork-arrest at the *RTS1*-RFB increased the frequency of replication slippage by only 2 times over spontaneous events: therefore, replication slippage occurs less frequently in survivors from recombination mutants than those from the wild-type strain (Figure 4D–4F). Based on 2DGE analysis, fork-restart is severely impaired in the absence of Rad22^Rad52^ (Figure 1B and [20]), such that even the two-fold induction in replication slippage by fork arrest in the *rad22-d* strain was surprising. The *rad22-d* strain accumulates suppressors involving the Fbh1 helicase that limits Rhp51^Rad51^- dependent recombination at blocked replication forks [50], [51]. Therefore, we analyzed replication slippage in the *rad22-d rhp51-d* double mutant in which no homologous recombination event occurs. In this background, there was no detectable fork-arrest-induced replication slippage (Figure 4D–4F). Thus, complete defect in fork restart results in a complete abolition of fork-arrest-induced replication slippage in the surviving population. Overall, our data establish that replication slippage results from inaccurate DNA synthesis during the restart of collapsed forks by recombination.

### Replication stress leading to fork collapse induces replication slippage

**Figure 5:**
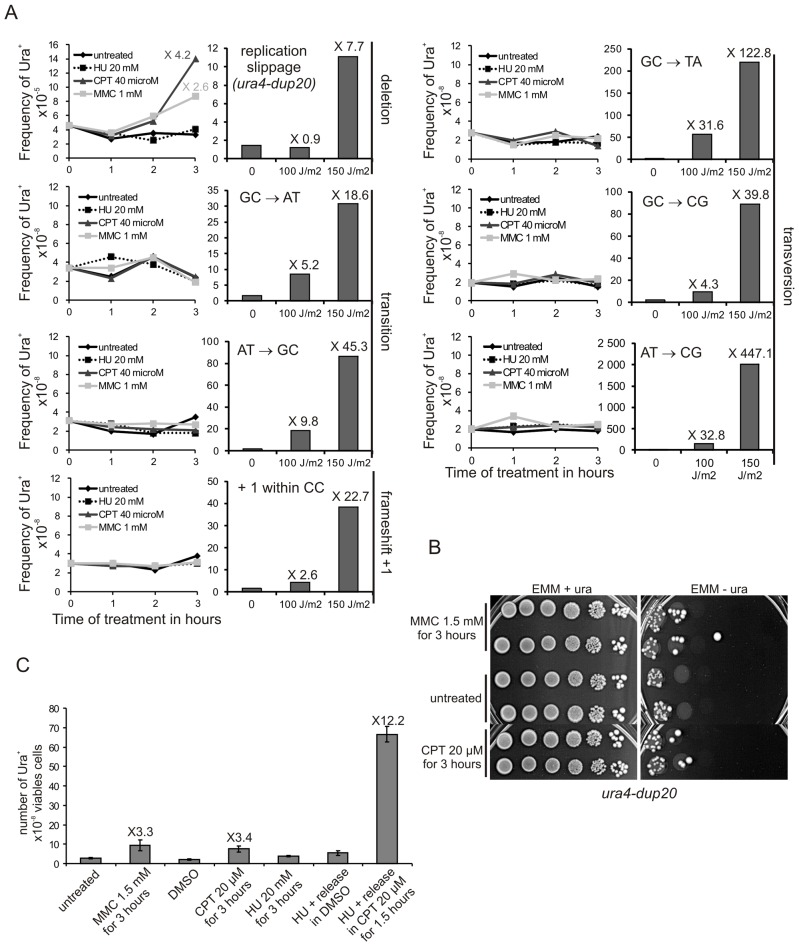
Collapsed forks, but not stalled forks, induce replication slippage. A. Left panel: the frequency of Ura^+^ revertants as a function of time-contact with indicated drugs for the indicated *ura4* alleles (single base-substitution, frame-shift, duplication of 20 nt). Right panel: the frequency of Ura^+^ revertants in response to UV-C irradiation as a function of dose for the indicated *ura4* alleles. The values reported are means of two independent experiments. Numbers indicate fold difference in the frequency of Ura^+^ revertants between the treated and untreated control samples. For *ura4* alleles containing base-substitutions or frame-shifts, the mutation event required to obtain Ura^+^ revertants is indicated on the figure. B. Serial tenfold-dilutions from *ura4-dup20* strain spotted onto the media indicated after treatment with MMC or CPT as indicated. C. Frequency of Ura^+^ revertants after the indicated treatments in the *ura4-dup20* strain. DMSO (the vehicle) was used as control for CPT treatment. The values reported are means of at least three independent experiments. Error bars correspond to SEM.

We investigated the effects of replication stress, other than the replication block imposed by the *RTS1*-RFB, on replication slippage. Strains harbouring *ura4^−^* alleles (base-substitutions, −1 frame-shift, and *ura4-dup20*) were exposed to replication-blocking agents or UV-C-induced DNA damages and the frequency of Ura^+^ revertants was scored. Three hours of treatment with either the topoisomerase I inhibitor camptothecin (CPT) or mitomycin C (MMC), an inter-strand cross-linking agent (ICls), increased the frequency of Ura^+^ revertants by 3 to 4 fold in the *ura4-dup20* strain (Figure 5A and 5B). At equivalent survival (70–90%), DNA-damages induced by a dose of 100 J/m^2^ of UV-C did not increase the frequency of Ura^+^ revertants in the *ura4-dup20* strain. Increasing the UV-C dose (150 J/m^2^) resulted in an increased reversion effect. The other *ura4* alleles exhibited an opposite behaviour pattern. As expected, UV-C-induced DNA damages, but not CPT or MMC treatment, increased the frequency of Ura^+^ revertants of the base-substitution and the −1 frame-shift mutants (Figure 5A). Thus, replication slippage, unlike other point mutations, appears to be a mutation event specifically induced by replication stress.

Hydroxyurea (HU) that prevents the bulk of dNTP synthesis during S-phase by inhibiting the ribonucleotide reductase, results in a slow-down of fork progression which did not induce replication slippage (Figure 5A). In contrast, CPT and MMC treatments that lead to replication stress by causing fork collapse induced replication slippage. Homologous recombination is repressed during HU treatment and recombination proteins are recruited to collapsed but not stalled forks [52]–[54]. Consistent with this, we found that the *rad22-d* mutant is highly sensitive to acute exposure to CPT, but not to HU (Figure S4). Thus, acute exposure to HU results in stalled forks that recover without recombination, whereas recombination may be required for restarting forks that have collapsed due to CPT or MMC treatment. We confirmed that CPT-induced replication slippage results from collapsed forks and was thus S-phase specific: the *ura4-dup20* strain was synchronized in early S-phase by HU treatment and released into S-phase with or without CPT. HU-synchronization and release into DMSO (used as vehicle for CPT) did not induce replication slippage. In contrast, the release of cells into S-phase in the presence of CPT stimulated replication slippage up to 12 fold (Figure 5C). These data indicate that CPT-induced fork collapse results in error-prone DNA synthesis characterized by replication slippage. These experiments further support the view that replication slippage results from recovery of collapsed forks by recombination and point out that the *RTS1*-barrier is representative of collapsed forks restarted by homologous recombination.

### TLS–DNA polymerases are not involved in fork-arrest-induced replication slippage

To investigate the inaccuracy of the DNA synthesis occurring immediately following the restart of collapsed forks, we analysed the involvement of TLS-DNA polymerases. In fission yeast, TLS pathways require either mono- or poly-ubiquitination of the clamp loader PCNA on lysine 164 [55]. We found that mutating this lysine to arginine residue did not affect replication slippage induced by the *RTS1-*RFB. None of Rev1, Rev3 or DinB DNA polymerases were required for fork-arrest-induced replication slippage (Figure S5). Therefore, the error-prone DNA synthesis associated with fork recovery by recombination does not rely on TLS DNA polymerases activity.

### The mismatch repair pathway does not counteract fork-arrest-induced replication slippage

The mismatch repair (MMR) pathway is temporally coupled to DNA replication, and MMR components are associated with replication centres [56]. The heterodimer Msh2/Msh6 recognises mispaired DNA and Msh2/Msh3 recognises small DNA loops up to 31 bases long, arising from replication slippage [57]. The failure to repair small DNA loops results in more frequent insertions and deletions [58]. Therefore, MMR activity could potentially lead to an underestimation of the extent of fork-arrest-induced replication slippage. However, replication slippage induced by the *RTS1*-RFB activity was as frequent in *msh2-d*, *msh6-d* and *msh3-d* strains as in wild-type control. Also, spontaneous replication slippage at *ura4* (without *RTS1*-RFB) was unaffected by the absence of MMR proteins (Figure S5). Therefore, there is no evidence that MMR repairs small DNA loops (20 nt) in fission yeast and fork-arrest-induced replication slippage is not counteracted by MMR in our model system.

## Discussion

**Figure 6:**
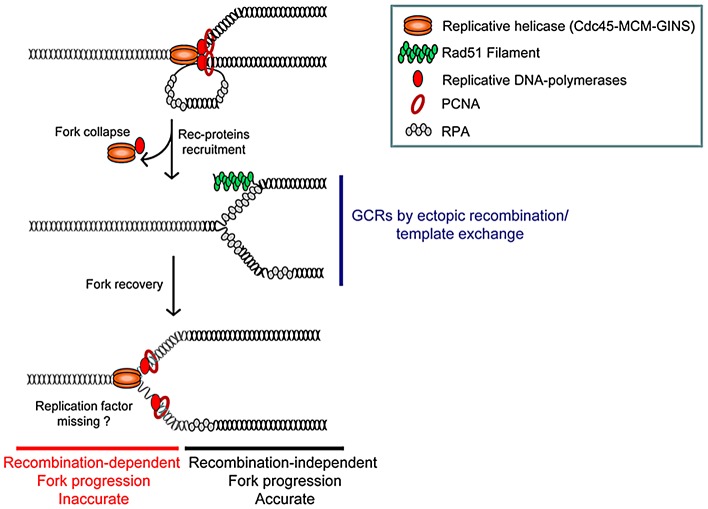
Model of replication-stress-induced genetic instability at collapsed forks. Collapsed forks might arise from torsional stress, fork breakage (*i.e.* at nick, ICLs), or proteins tightly-bound to DNA. Replisome disassembly at collapsed forks may favour the unwinding of the nascent strand on which Rad51 nucleates. At this initial stage of fork resumption by recombination, homology-driven template exchange can promote intra- or inter-recombination resulting in GCRs. Fork recovery by recombination overcomes the initial replication block and allows an inaccurate replisome to form (see text for details).

Using conditional fork arrest constructs, we studied the consequences for genome instability of impediments to replication forks progression. A single fork arrest results in large-scale genomic changes and mutations that occur during recombination-mediated fork recovery (Figure 6). Inappropriate ectopic recombination at arrested forks results in GCRs, whereas appropriate restarting of the fork on the initial template results in error-prone DNA synthesis. GCRs and mutations at collapsed forks are genetically separable: Rqh1 limits fork-arrest-induced GCRs but not mutations (Figure 2D and Figure 3B). We demonstrate here that collapsed forks whose progression resumes by recombination lose accuracy during DNA synthesis, resulting in frequent intra-template switches. Thus, homologous recombination contributes to completion of DNA replication when forks progression is impeded but also fuels genome modifications both at the chromosomal and nucleotide level.

Non allelic homologous recombination (NAHR) between low copy number repeats (LCR) contributes to recombination-mediated GCRs in mitosis and meiosis. NAHR is responsible for translocations, deletions, inversions and loss of heterozygosity [59]. Ou *et al.* predicted 1143 LCR pairs in the human genome liable to mediate recurrent translocations [60]. In budding yeast, a single DSB is sufficient to mediate recombination-dependent translocation [61]. Here, we report that a single collapsed fork increases the rate of genomic deletion 40 fold, and that of translocation 5 fold. Fork-arrest-induced GCRs are mediated by NAHR between heterologous chromosomes. It is not clear whether fork arrest on both homologous repeats contributes to fork-arrest-induced GCRs. We could not address this question in our model system, because the *RTS1* sequence close to the *mat1* locus on chromosome II has a low RFB activity [62]. Also, the recruitment of recombination proteins at the *RTS1* sequence near the *mat1* locus is not regulated by the level of Rtf1 expression, showing that regulating Rtf1 expression was in itself insufficient to regulate the *RTS1*-RFB activity at the *mat1* locus [35].

Inverted repeats (IRs) are structural elements that contribute to genome instability. Impediments to replication forks progressing through IRs favour their fusion to generate acentric and dicentric inverted chromosomes [11], [14]. IRs in humans can also trigger the formation of inverted genomic segments and complex triplication rearrangements by a replication-based mechanism [47]. Here, we report that IRs near collapsed forks can increase the rate of GCRs by up to 1,500 fold. This high level of GCRs cannot be explained merely by the addition of independent inter- and intra-chromosomal recombination events. Rather, our analyses suggest that IRs may stimulate tri-parental recombination events induced by template switching of nascent strands at collapsed forks, such that three homologous sequences are involved. Similarly, recombination-dependent translocations induced by a single DSB in budding yeast is proposed to be the consequence of tri-parental recombination [63], [64]. One possible mechanism is that IRs-induced GCRs result from successive template switches initiated by nascent strands at collapsed forks, reminiscent of the multiple template switches during break-induced-replication (BIR) in budding yeast [33]. Interestingly, Rqh1 prevents fork-arrest-induced GCRs by limiting both inter- and intra-chromosomal recombination without affecting fork restart efficiency. Thus, tri-parental recombination might correspond to multiple and successive template switches between homologous repeats.

The high accuracy of DNA replication is compromised by impediments to fork progression, and recombination-mediated fork recovery results in decreased processivity of DNA synthesis (Figure 6). Recombination-induced mutations associated with DSBs or impeding DNA replication have been described previously. The formation of damaged single-stranded DNA during the resection of DSBs favours base-substitutions [65]. We detected the recruitment of the single-strand binding protein RPA up to 1.4 kb behind, but not ahead of the *RTS1*-arrested fork (data to be published), showing that fork-arrest-induced mutation is not correlated with damaged single-stranded DNA exposed behind collapsed forks. Nevertheless, there were rare replication slippage occurred behind *RTS1*-arrested forks (in the *t<ura4-ori* construct), suggesting that resumption of DNA synthesis can in some cases occur at a position behind the site of the collapsed fork. Recombination-dependent DNA synthesis occurring outside S-phase is also highly inaccurate during gene conversion and BIR, resulting in either template switches, base-substitutions or frame-shifts [33], [34], [66]. Elevated dNTP pools, due to activation of the DNA-damage checkpoint in G2 cells, contributes to the generation of mutations when hundreds of kbs are synthesised during BIR [34]. Here, we demonstrate that during normal S-phase progression, a single collapsed fork, restored by recombination, results in replication slippage up to 1.2 Kb away from the initial restarting point.

Recombination-induced replication slippage has been reported previously.

In fission yeast, a defect in pol alpha (*swi7-H4* mutant) is associated with a recombination-mediated mutator phenotype characterized by an increased frequency of base-substitutions and Del/Dup between short direct repeats [67]. DNA-polymerase kappa (DinB) and zeta (Rev3) are responsible for the increased base-substitution rate, but the DNA-polymerases involved in Del/Dup mutations were not identified [68]. In budding yeast, a defect in polymerase delta (*pol3-t* mutant) results in an increased level of replication slippage, mediated by homologous recombination [49]. In contrast, the increased rate of replication slippage in the absence of the accessory subunit of polymerase delta, Pol32, does not depend on a functional recombination pathway [69]. Here, we report that recovery of collapsed forks by recombination is specifically associated with replication slippage. Nonetheless, spontaneous replication slippage events are also increased in strains mutated for recombination genes (Figure 4D–4F). Pol32 is required for BIR and replication-induced template switches leading to segmental duplication [70], [71]. Recombination is responsible for only half of these segmental duplications. Thus, it is possible that fork-restart mechanisms dependent on Pol32 and homologous recombination are prone to replication slippage and that in the absence of these pathways alternative micro-homology mediated mechanisms are revealed.

We suggest that at least two steps of the recombination-based fork recovery mechanism can compromise genome stability (Figure 6). At an initial stage, recruitment of recombination proteins on stalled nascent strands favours both fork recovery and ectopic template switches leading to GCRs. At a later stage, once the replisome has been reconstituted and the fork has resumed its progression, the nascent strands are prone to intra-template switching leading to replication slippage. The reasons for the inaccuracy of DNA synthesis associated with restarted forks during scheduled DNA replication (*i.e.* in S-phase) remain to be determined. One possibility is that one or more factors are missing in the rebuilt replisome during recovery by recombination. Oncogene-induced replication stress results from unbalanced DNA replication that contributes to genome instability in precancerous cells [12]. Completion of DNA replication in such stress conditions is likely to rely on recombination-mediated fork recovery that in turn generates genome instability. Insertions/deletions flanked by micro-homology, responsible for copy number variations (CNVs), have been identified both in cancer cells and also in response to replication inhibition [72], [73]; their reported sizes are between 1 Kb and several tens of mega bases, but the analysis of these features has been limited by the resolution of array-based genomics approaches. Sub-microscopic insertions/deletions flanked by micro-homology have been also described at loci in which replication origins are scarce, including the human fragile site FRA3B, the instability of which is a consequence of replication stress [74]. Interestingly, homologous recombination contributes to the stability of fragile sites by facilitating complete replication or by repairing gaps and breaks at these sites. Thus, we propose that micro-homology-mediated CNVs could be viewed as scars left by error-prone replication forks restarted by recombination.

## Materials and Methods

### Standard genetic and molecular biology

Strains used were constructed by standard genetic techniques and are listed in Table S1. 2DGE was performed as previously described [20]. To create *ura4-dup20* and *ura4-dup22* alleles associated or not with the *RTS1*-RFB, genomic DNA was isolated from selected 5-FOA^R^ cells containing duplications identified by sequencing. A PCR fragment containing duplications within the *ura4* ORF was amplified using the following primers: TTCTGTTCCAACA-CCAATGTTT and TCACGTTTATTTTCAAACATCCA. The PCR products were purified and used to transform strains SL206 (*ura4^+^*), SL350 (*t-ura4<ori*) and SL504 (*t<ura4-ori*). Transformants were selected on 5-FOA-containing plates. Appropriate replacement of *ura4+* by *ura4-dup20* or *ura4-dup22* was verified by PCR and sequencing.

### *ura4* loss assay

A minimum of 11 independent single colonies from appropriate strains growing with or without thiamine were inoculated in 10 ml of non-selective media (with or without thiamine) and grown to stationary phase. Appropriate dilutions were plated on supplemented YEA to determine plating efficiency and on 5-FOA-containing plates. Colonies were counted after 5–7 days of incubation at 30°C. The rate of *ura4* loss was determined with the method of the median and data are presented on Table 1. Statistical significance was detected using the nonparametric Mann-Whitney U test.

### PCR assays and sequencing to determine the rates of genomic deletion, translocation, and mutation

At least 200 5-FOA^R^ colonies per strain and condition were subjected to PCR analysis with the following primers: AAAACAAACGCAAACAAGGC and GTTTAACTATGCTTCGTCGG to amplify *ura4* ORF, TGAATCCTCCGTTCAGTAGG and AAGGACTGCGTTCTTCTAGC to amplify *rng3* and TTTCCTTTCACGGCTAACCC (TLII) and TGTACCCATGAGCAAACTGC (TLIII) to amplify the translocation junction. The amplified *ura4* fragments were then sequenced on both strands, with the primers used to amplify the *ura4* ORF. Only mutations present on both strands were considered to determine mutation spectra. Deletions, mutations and translocations were scored as percentages of all events and these values were used to balance the rates of *ura4* loss to determine the respective rates of deletion, mutation and translocation (see Figure S1 for deletion and translocation rates and Figure S2 for mutation rates).

The fork-arrest-induced deletion, translocation and mutation rates (Figure 2C–2E and Figure 3A) were calculated by subtracting the rate obtained in presence of thiamine (Rtf1 being repressed, OFF) from the rate obtained in the absence of thiamine (Rtf1 being expressed, ON). This method allows the spontaneous instability of IRs and the leakiness of the *RTS1*-RFB activity to be disregarded to determine strictly the rate of events induced by fork-arrest. The nonparametric Mann-Whitney U test was used to test for statistically significant differences.

### Reverse Mutation Assay

Exponentially growing cells were treated with 20 mM of HU, 40 µM of CPT or 1 mM of MMC. At indicated times, samples were taken and appropriate dilutions were plated on supplemented minimal media to determine plating efficiency and on uracil-free plates. Colonies were counted after incubation at 30°C for 5–7 days and the frequency of Ura^+^ colonies was determined.

### Replication Slippage Assay Using *Ura4-Dup20* And *Ura4-Dup22* Strains

For strains showing a slow growth phenotype (recombination mutants), the frequency of Ura^+^ revertants was determined as a function of the number of generations experiencing fork arrest at *ura4*. Exponentially growing 5-FOA^R^ cells were washed twice in water and used to inoculate uracil-containing media without thiamine. Every 24 hours, cells were counted to determine the number of generations, and appropriate dilutions were plated on supplemented minimal media and on uracil-free plates. Colonies were counted after incubation at 30°C for 5–7 days and the frequency of Ura^+^ colonies was determined. The slope of the curves presented on Figure 4F corresponds to the rate of replication slippage/generation. For strains showing similar growth to wild-type cells, a single 5-FOA^R^ colony was grown on uracil-containing plates with or without thiamine for 2–3 days, and then grown in uracil-containing media with or without thiamine for 2 days at 30°C. Appropriate dilutions were plated on supplemented minimal media and on uracil-free plates. Colonies were counted after incubation at 30°C for 5–7 days and the frequency of Ura^+^ colonies was determined.

## Supporting Information

Figure S1 Fork-arrest results in GCRs in a recombination-dependent manner. A. The rate of deletion for indicated strains, in the presence (Rtf1 repressed) and in the absence (Rtf1 expressed) of thiamine. Numbers of *RTS1* repeats present in the *S. pombe* genome and the presence of a visible fork arrest (based on 2DGE presented on Figure 1B) are given for each strain. The % of deletion events, as determined by the PCR assay, was used to balance the rate of *ura4* loss. The values reported are means of at least 3 independent median rates. Error bars correspond to the standard error (SE). Statistically significant fold differences in the rate of deletion events between the Rtf1 “repressed” and “expressed” conditions are indicated with an *. B and C. Rate of deletion (B) and translocation (C) for the strains indicated; ON and OFF refers to the *RTS1*-RFB being active or not, respectively. The % of deletion and translocation events, as determined by the PCR assay, was used to balance the rate of *ura4* loss. The values reported are means at least 3 independent median rates. Error bars correspond to SE. Statistically significant fold differences in the rates of deletion or translocation events between the “OFF” and “ON” conditions are indicated with an *. Translocation events (based on the detection of the TLII/TLIII PCR product) were not detected in *rad22-d* or *rhp51-d* strains, whatever the conditional fork arrest construct.
(TIF)

Figure S2 Fork-arrest induces replication slippage. A. The rate of mutation for indicated strains, in the presence (Rtf1 repressed) and in the absence (Rtf1 expressed) of thiamine. Numbers of *RTS1* repeats present in the *S. pombe* genome and the presence of a visible fork arrest (based on 2DGE presented on Figure 1) are given for each strain. The % of mutation events, as determined by the PCR assay and sequencing, was used to balance the rate of *ura4* loss. The reported values are means of at least 3 independent median rates. Error bars correspond to SE. Statistically significant fold differences in the rate of mutation events between the Rtf1 “repressed” and “expressed” conditions are indicated with an *. B. The frequency of Ura^+^ revertants for the indicated strains and conditions. All strains harbour a non-functional *ura4* allele due to a single base-substitution or a frame-shift or a duplication of 20 or 22 nt, together with the *RTS1*-RFB in the *t-ura4<ori* context. The initial mutations and expected reverted mutations are indicated in the table. #1 and #2 correspond to two independent mutated strains for each type of mutation.
(TIF)

Figure S3 Features of replication slippage induced by fork arrest. A. Table of deletion/duplication and micro-homology features. B. Map of deletion and duplication events observed within the *ura4* ORF in the *t-ura4<ori* construct upon fork arrest. Del and Dup stand for deletion and duplication, respectively.
(TIF)

Figure S4 Sensitivity of *rad22-d* strain to acute exposure to 20 mM of HU or 20 µM of CPT. The values reported are means of two to four independent experiments. Error bars indicate the standard error of the mean (SEM).
(TIF)

Figure S5 Fork-arrest-induced replication slippage is independent of the post-replication repair and mismatch repair. A–C. Left panels: Serial tenfold-dilutions of indicated strains cultured in thiamine-free medium spotted onto the medium indicated. *RTS1*-RFB activity “–” refers to the strain *t-ura4-dup20-ori* and “+” refers to the strain *t-ura4-dup20<ori*. Right panels: The frequency of Ura^+^ revertants from the strains indicated (*t-ura4-dup20-ori* associated or not with the *RTS1*-RFB) in the conditions indicated. The values reported are means of at least three independent experiments and error bars correspond to SEM.
(TIF)

Table S1 Strains used in this study.
(DOCX)

## References

[1] Aguilera A, Gomez-Gonzalez B (2008) Genome instability: a mechanistic view of its causes and consequences. Nat Rev Genet 9: 204–217. 18227811 10.1038/nrg2268

[2] Branzei D, Foiani M (2010) Maintaining genome stability at the replication fork. Nat Rev Mol Cell Biol 11: 208–219. 20177396 10.1038/nrm2852

[3] Halazonetis TD, Gorgoulis VG, Bartek J (2008) An oncogene-induced DNA damage model for cancer development. Science 319: 1352–1355. 18323444 10.1126/science.1140735

[4] Zhang F, Carvalho CM, Lupski JR (2009) Complex human chromosomal and genomic rearrangements. Trends Genet 25: 298–307. 19560228 10.1016/j.tig.2009.05.005 PMC4464790

[5] Letessier A, Millot GA, Koundrioukoff S, Lachages AM, Vogt N, et al (2011) Cell-type-specific replication initiation programs set fragility of the FRA3B fragile site. Nature 470: 120–123. 21258320 10.1038/nature09745

[6] Le Tallec B, Dutrillaux B, Lachages AM, Millot GA, Brison O, et al (2011) Molecular profiling of common fragile sites in human fibroblasts. Nat Struct Mol Biol 18: 1421–1423. 22056772 10.1038/nsmb.2155

[7] Petermann E, Helleday T (2010) Pathways of mammalian replication fork restart. Nat Rev Mol Cell Biol 11: 683–687. 20842177 10.1038/nrm2974

[8] Hastings PJ, Ira G, Lupski JR (2009) A microhomology-mediated break-induced replication model for the origin of human copy number variation. PLoS Genet 5: e1000327 doi:10.1371/journal.pgen.1000327 19180184 10.1371/journal.pgen.1000327 PMC2621351

[9] Weinert T, Kaochar S, Jones H, Paek A, Clark AJ (2009) The replication fork's five degrees of freedom, their failure and genome rearrangements. Curr Opin Cell Biol 21: 778–784. 19913398 10.1016/j.ceb.2009.10.004

[10] Lemoine FJ, Degtyareva NP, Lobachev K, Petes TD (2005) Chromosomal translocations in yeast induced by low levels of DNA polymerase a model for chromosome fragile sites. Cell 120: 587–598. 15766523 10.1016/j.cell.2004.12.039

[11] Mizuno K, Lambert S, Baldacci G, Murray JM, Carr AM (2009) Nearby inverted repeats fuse to generate acentric and dicentric palindromic chromosomes by a replication template exchange mechanism. Genes Dev 23: 2876–2886. 20008937 10.1101/gad.1863009 PMC2800087

[12] Bester AC, Roniger M, Oren YS, Im MM, Sarni D, et al (2011) Nucleotide deficiency promotes genomic instability in early stages of cancer development. Cell 145: 435–446. 21529715 10.1016/j.cell.2011.03.044 PMC3740329

[13] Ozeri-Galai E, Lebofsky R, Rahat A, Bester AC, Bensimon A, et al (2011) Failure of origin activation in response to fork stalling leads to chromosomal instability at fragile sites. Mol Cell 43: 122–131. 21726815 10.1016/j.molcel.2011.05.019

[14] Paek AL, Kaochar S, Jones H, Elezaby A, Shanks L, et al (2009) Fusion of nearby inverted repeats by a replication-based mechanism leads to formation of dicentric and acentric chromosomes that cause genome instability in budding yeast. Genes Dev 23: 2861–2875. 20008936 10.1101/gad.1862709 PMC2800083

[15] Blow JJ, Ge XQ, Jackson DA (2011) How dormant origins promote complete genome replication. Trends Biochem Sci 36: 405–414. 21641805 10.1016/j.tibs.2011.05.002 PMC3329722

[16] Mirkin EV, Mirkin SM (2007) Replication fork stalling at natural impediments. Microbiol Mol Biol Rev 71: 13–35. 17347517 10.1128/MMBR.00030-06 PMC1847372

[17] Lambert S, Froget B, Carr AM (2007) Arrested replication fork processing: interplay between checkpoints and recombination. DNA Repair (Amst) 6: 1042–1061. 17412649 10.1016/j.dnarep.2007.02.024

[18] Kawabata T, Luebben SW, Yamaguchi S, Ilves I, Matise I, et al (2011) Stalled fork rescue via dormant replication origins in unchallenged S phase promotes proper chromosome segregation and tumor suppression. Mol Cell 41: 543–553. 21362550 10.1016/j.molcel.2011.02.006 PMC3062258

[19] Murray JM, Carr AM (2008) Smc5/6: a link between DNA repair and unidirectional replication? Nat Rev Mol Cell Biol 9: 177–182. 18059412 10.1038/nrm2309

[20] Lambert S, Mizuno K, Blaisonneau J, Martineau S, Chanet R, et al (2010) Homologous recombination restarts blocked replication forks at the expense of genome rearrangements by template exchange. Mol cell 39: 346–359. 20705238 10.1016/j.molcel.2010.07.015

[21] Katou Y, Kanoh Y, Bando M, Noguchi H, Tanaka H, et al (2003) S-phase checkpoint proteins Tof1 and Mrc1 form a stable replication-pausing complex. Nature 424: 1078–1083. 12944972 10.1038/nature01900

[22] De Piccoli G, Katou Y, Itoh T, Nakato R, Shirahige K, et al (2012) Replisome stability at defective DNA replication forks is independent of s phase checkpoint kinases. Mol Cell 45: 696–704. 22325992 10.1016/j.molcel.2012.01.007

[23] Froget B, Blaisonneau J, Lambert S, Baldacci G (2008) Cleavage of stalled forks by fission yeast Mus81/Eme1 in absence of DNA replication checkpoint. Mol Biol Cell 19: 445–456. 18032583 10.1091/mbc.E07-07-0728 PMC2230577

[24] Cotta-Ramusino C, Fachinetti D, Lucca C, Doksani Y, Lopes M, et al (2005) Exo1 processes stalled replication forks and counteracts fork reversal in checkpoint-defective cells. Mol Cell 17: 153–159. 15629726 10.1016/j.molcel.2004.11.032

[25] Heller RC, Marians KJ (2006) Replisome assembly and the direct restart of stalled replication forks. Nat Rev Mol Cell Biol 7: 932–943. 17139333 10.1038/nrm2058

[26] Michel B, Boubakri H, Baharoglu Z, LeMasson M, Lestini R (2007) Recombination proteins and rescue of arrested replication forks. DNA Repair (Amst) 6: 967–980. 17395553 10.1016/j.dnarep.2007.02.016

[27] Hashimoto Y, Puddu F, Costanzo V (2011) RAD51- and MRE11-dependent reassembly of uncoupled CMG helicase complex at collapsed replication forks. Nat Struct Mol Biol 19: 17–24. 22139015 10.1038/nsmb.2177 PMC4306020

[28] Roseaulin L, Yamada Y, Tsutsui Y, Russell P, Iwasaki H, et al (2008) Mus81 is essential for sister chromatid recombination at broken replication forks. Embo J 27: 1378–1387. 18388861 10.1038/emboj.2008.65 PMC2374842

[29] Moriel-Carretero M, Aguilera A (2010) A postincision-deficient TFIIH causes replication fork breakage and uncovers alternative Rad51- or Pol32-mediated restart mechanisms. Mol Cell 37: 690–701. 20227372 10.1016/j.molcel.2010.02.008

[30] Lydeard JR, Lipkin-Moore Z, Sheu YJ, Stillman B, Burgers PM, et al (2010) Break-induced replication requires all essential DNA replication factors except those specific for pre-RC assembly. Genes Dev 24: 1133–1144. 20516198 10.1101/gad.1922610 PMC2878651

[31] Llorente B, Smith CE, Symington LS (2008) Break-induced replication: what is it and what is it for? Cell Cycle 7: 859–864. 18414031 10.4161/cc.7.7.5613

[32] McEachern MJ, Haber JE (2006) Break-induced replication and recombinational telomere elongation in yeast. Annu Rev Biochem 75: 111–135. 16756487 10.1146/annurev.biochem.74.082803.133234

[33] Smith CE, Llorente B, Symington LS (2007) Template switching during break-induced replication. Nature 447: 102–105. 17410126 10.1038/nature05723

[34] Deem A, Keszthelyi A, Blackgrove T, Vayl A, Coffey B, et al (2011) Break-induced replication is highly inaccurate. PLoS Biol 9: e1000594 doi:10.1371/journal.pbio.1000594 21347245 10.1371/journal.pbio.1000594 PMC3039667

[35] Lambert S, Watson A, Sheedy DM, Martin B, Carr AM (2005) Gross chromosomal rearrangements and elevated recombination at an inducible site-specific replication fork barrier. Cell 121: 689–702. 15935756 10.1016/j.cell.2005.03.022

[36] Eydmann T, Sommariva E, Inagawa T, Mian S, Klar AJ, et al (2008) Rtf1-mediated eukaryotic site-specific replication termination. Genetics 180: 27–39. 18723894 10.1534/genetics.108.089243 PMC2535681

[37] Kaplan DL, Bastia D (2009) Mechanisms of polar arrest of a replication fork. Mol Microbiol 72: 279–285. 19298368 10.1111/j.1365-2958.2009.06656.x

[38] McInerney P, O'Donnell M (2007) Replisome fate upon encountering a leading strand block and clearance from DNA by recombination proteins. J Biol Chem 282: 25903–25916. 17609212 10.1074/jbc.M703777200

[39] Sabouri N, McDonald KR, Webb CJ, Cristea IM, Zakian VA (2012) DNA replication through hard-to-replicate sites, including both highly transcribed RNA Pol II and Pol III genes, requires the S. pombe Pfh1 helicase. Genes Dev 26: 581–593. 22426534 10.1101/gad.184697.111 PMC3315119

[40] Steinacher R, Osman F, Dalgaard JZ, Lorenz A, Whitby MC (2012) The DNA helicase Pfh1 promotes fork merging at replication termination sites to ensure genome stability. Genes Dev 26: 594–602. 22426535 10.1101/gad.184663.111 PMC3315120

[41] Branzei D, Vanoli F, Foiani M (2008) SUMOylation regulates Rad18-mediated template switch. Nature 456: 915–920. 19092928 10.1038/nature07587

[42] Miyabe I, Kunkel TA, Carr AM (2011) The major roles of DNA polymerases epsilon and delta at the eukaryotic replication fork are evolutionarily conserved. PLoS Genet 7: e1002407 doi:10.1371/journal.pgen.1002407 22144917 10.1371/journal.pgen.1002407 PMC3228825

[43] Myung K, Chen C, Kolodner RD (2001) Multiple pathways cooperate in the suppression of genome instability in Saccharomyces cerevisiae. Nature 411: 1073–1076. 11429610 10.1038/35082608

[44] Chen C, Kolodner RD (1999) Gross chromosomal rearrangements in Saccharomyces cerevisiae replication and recombination defective mutants. Nat Genet 23: 81–85. 10471504 10.1038/12687

[45] Putnam CD, Hayes TK, Kolodner RD (2009) Specific pathways prevent duplication-mediated genome rearrangements. Nature 460: 984–989. 19641493 10.1038/nature08217 PMC2785216

[46] Voineagu I, Narayanan V, Lobachev KS, Mirkin SM (2008) Replication stalling at unstable inverted repeats: interplay between DNA hairpins and fork stabilizing proteins. Proc Natl Acad Sci U S A 105: 9936–9941. 18632578 10.1073/pnas.0804510105 PMC2481305

[47] Carvalho CM, Ramocki MB, Pehlivan D, Franco LM, Gonzaga-Jauregui C, et al (2011) Inverted genomic segments and complex triplication rearrangements are mediated by inverted repeats in the human genome. Nat Genet 43: 1074–1081. 21964572 10.1038/ng.944 PMC3235474

[48] Lichten M, Haber JE (1989) Position effects in ectopic and allelic mitotic recombination in Saccharomyces cerevisiae. Genetics 123: 261–268. 2684745 10.1093/genetics/123.2.261 PMC1203798

[49] Tran HT, Degtyareva NP, Koloteva NN, Sugino A, Masumoto H, et al (1995) Replication slippage between distant short repeats in Saccharomyces cerevisiae depends on the direction of replication and the RAD50 and RAD52 genes. Mol Cell Biol 15: 5607–5617. 7565712 10.1128/mcb.15.10.5607 PMC230811

[50] Lorenz A, Osman F, Folkyte V, Sofueva S, Whitby MC (2009) Fbh1 limits Rad51-dependent recombination at blocked replication forks. Mol Cell Biol 29: 4742–4756. 19546232 10.1128/MCB.00471-09 PMC2725720

[51] Osman F, Dixon J, Barr AR, Whitby MC (2005) The F-Box DNA helicase Fbh1 prevents Rhp51-dependent recombination without mediator proteins. Mol Cell Biol 25: 8084–8096. 16135800 10.1128/MCB.25.18.8084-8096.2005 PMC1234329

[52] Alabert C, Bianco JN, Pasero P (2009) Differential regulation of homologous recombination at DNA breaks and replication forks by the Mrc1 branch of the S-phase checkpoint. Embo J 28: 1131–1141. 19322196 10.1038/emboj.2009.75 PMC2683710

[53] Lisby M, Barlow JH, Burgess RC, Rothstein R (2004) Choreography of the DNA damage response: spatiotemporal relationships among checkpoint and repair proteins. Cell 118: 699–713. 15369670 10.1016/j.cell.2004.08.015

[54] Meister P, Taddei A, Vernis L, Poidevin M, Gasser SM, et al (2005) Temporal separation of replication and recombination requires the intra-S checkpoint. J Cell Biol 168: 537–544. 15716375 10.1083/jcb.200410006 PMC2171758

[55] Coulon S, Ramasubramanyan S, Alies C, Philippin G, Lehmann A, et al (2010) Rad8Rad5/Mms2-Ubc13 ubiquitin ligase complex controls translesion synthesis in fission yeast. Embo J 29: 2048–2058. 20453833 10.1038/emboj.2010.87 PMC2892369

[56] Hombauer H, Campbell CS, Smith CE, Desai A, Kolodner RD (2011) Visualization of eukaryotic DNA mismatch repair reveals distinct recognition and repair intermediates. Cell 147: 1040–1053. 22118461 10.1016/j.cell.2011.10.025 PMC3478091

[57] Tran HT, Gordenin DA, Resnick MA (1996) The prevention of repeat-associated deletions in Saccharomyces cerevisiae by mismatch repair depends on size and origin of deletions. Genetics 143: 1579–1587. 8844147 10.1093/genetics/143.4.1579 PMC1207422

[58] Sia EA, Jinks-Robertson S, Petes TD (1997) Genetic control of microsatellite stability. Mutat Res 383: 61–70. 9042420 10.1016/s0921-8777(96)00046-8

[59] Liu P, Carvalho CM, Hastings P, Lupski JR (2012) Mechanisms for recurrent and complex human genomic rearrangements. Curr Opin Genet Dev 10.1016/j.gde.2012.02.012 PMC3378805 22440479

[60] Ou Z, Stankiewicz P, Xia Z, Breman AM, Dawson B, et al (2010) Observation and prediction of recurrent human translocations mediated by NAHR between nonhomologous chromosomes. Genome Res 21: 33–46. 10.1101/gr.111609.110 PMC3012924 21205869

[61] Bosco G, Haber JE (1998) Chromosome break-induced DNA replication leads to nonreciprocal translocations and telomere capture. Genetics 150: 1037–1047. 9799256 10.1093/genetics/150.3.1037 PMC1460379

[62] Dalgaard JZ, Klar AJ (2001) A DNA replication-arrest site RTS1 regulates imprinting by determining the direction of replication at mat1 in S. pombe. Genes Dev 15: 2060–2068. 11511538 10.1101/gad.200801 PMC312760

[63] Ruiz JF, Gomez-Gonzalez B, Aguilera A (2009) Chromosomal translocations caused by either pol32-dependent or pol32-independent triparental break-induced replication. Mol Cell Biol 29: 5441–5454. 19651902 10.1128/MCB.00256-09 PMC2756893

[64] Schmidt KH, Wu J, Kolodner RD (2006) Control of translocations between highly diverged genes by Sgs1, the Saccharomyces cerevisiae homolog of the Bloom's syndrome protein. Mol Cell Biol 26: 5406–5420. 16809776 10.1128/MCB.00161-06 PMC1592713

[65] Yang Y, Sterling J, Storici F, Resnick MA, Gordenin DA (2008) Hypermutability of damaged single-strand DNA formed at double-strand breaks and uncapped telomeres in yeast Saccharomyces cerevisiae. PLoS Genet 4: e1000264 doi:10.1371/journal.pgen.1000264 19023402 10.1371/journal.pgen.1000264 PMC2577886

[66] Hicks WM, Kim M, Haber JE (2010) Increased mutagenesis and unique mutation signature associated with mitotic gene conversion. Science 329: 82–85. 20595613 10.1126/science.1191125 PMC4254764

[67] Kai M, Boddy MN, Russell P, Wang TS (2005) Replication checkpoint kinase Cds1 regulates Mus81 to preserve genome integrity during replication stress. Genes Dev 19: 919–932. 15805465 10.1101/gad.1304305 PMC1080131

[68] Kai M, Wang TS (2003) Checkpoint activation regulates mutagenic translesion synthesis. Genes Dev 17: 64–76. 12514100 10.1101/gad.1043203 PMC195967

[69] Huang ME, de Calignon A, Nicolas A, Galibert F (2000) POL32, a subunit of the Saccharomyces cerevisiae DNA polymerase delta, defines a link between DNA replication and the mutagenic bypass repair pathway. Curr Genet 38: 178–187. 11126776 10.1007/s002940000149

[70] Payen C, Koszul R, Dujon B, Fischer G (2008) Segmental duplications arise from Pol32-dependent repair of broken forks through two alternative replication-based mechanisms. PLoS Genet 4: e1000175 doi:10.1371/journal.pgen.1000175 18773114 10.1371/journal.pgen.1000175 PMC2518615

[71] Lydeard JR, Jain S, Yamaguchi M, Haber JE (2007) Break-induced replication and telomerase-independent telomere maintenance require Pol32. Nature 448: 820–823. 17671506 10.1038/nature06047

[72] Colnaghi R, Carpenter G, Volker M, O'Driscoll M (2011) The consequences of structural genomic alterations in humans: Genomic Disorders, genomic instability and cancer. Semin Cell Dev Biol 22: 875–885. 21802523 10.1016/j.semcdb.2011.07.010

[73] Arlt MF, Wilson TE, Glover TW (2012) Replication stress and mechanisms of CNV formation. Curr Opin Genet Dev 10.1016/j.gde.2012.01.009 PMC3371136 22365495

[74] Durkin SG, Ragland RL, Arlt MF, Mulle JG, Warren ST, et al (2008) Replication stress induces tumor-like microdeletions in FHIT/FRA3B. Proc Natl Acad Sci U S A 105: 246–251. 18162546 10.1073/pnas.0708097105 PMC2224195

