# Supplementary figures and images for: Recovery of Arrested Replication Forks by Homologous Recombination Is Error-Prone
Source: PLoS Genet. 2012 Oct 18;8(10):e1002976. doi: 10.1371/journal.pgen.1002976 (PMC3475662; doi:10.1371/journal.pgen.1002976)

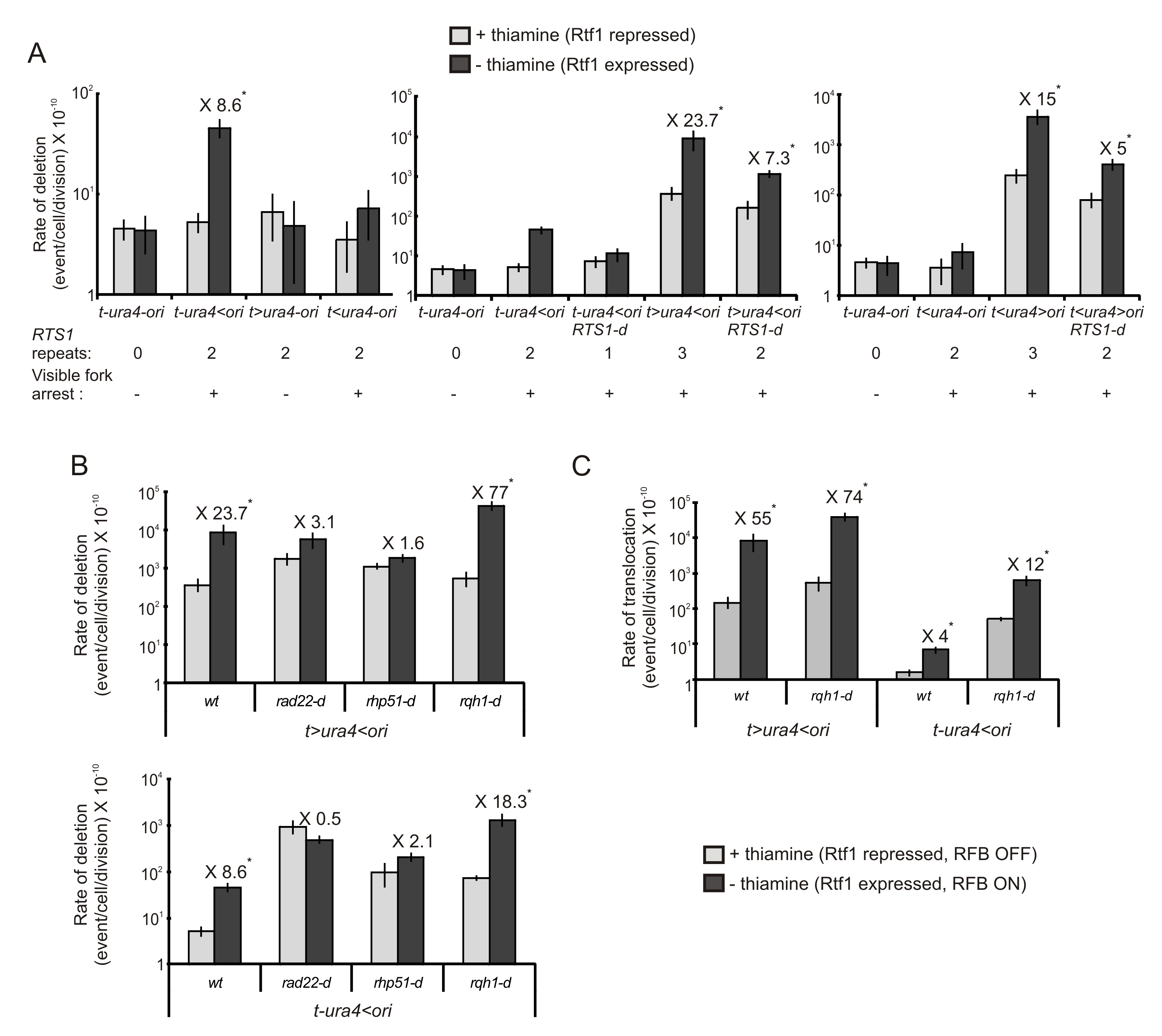

Supplement: Figure S1 — Fork-arrest results in GCRs in a recombination-dependent manner. A. The rate of deletion for indicated strains, in the presence (Rtf1 repressed) and in the absence (Rtf1 expressed) of thiamine. Numbers of RTS1 repeats present in the S. pombe genome and the presence of a visible fork arrest (based on 2DGE presented on Figure 1B) are given for each strain. The % of deletion events, as determined by the PCR assay, was used to balance the rate of ura4 loss. The values reported are means of at least 3 independent median rates. Error bars correspond to the standard error (SE). Statistically significant fold differences in the rate of deletion events between the Rtf1 “repressed” and “expressed” conditions are indicated with an *. B and C. Rate of deletion (B) and translocation (C) for the strains indicated; ON and OFF refers to the RTS1-RFB being active or not, respectively. The % of deletion and translocation events, as determined by the PCR assay, was used to balance the rate of ura4 loss. The values reported are means at least 3 independent median rates. Error bars correspond to SE. Statistically significant fold differences in the rates of deletion or translocation events between the “OFF” and “ON” conditions are indicated with an *. Translocation events (based on the detection of the TLII/TLIII PCR product) were not detected in rad22-d or rhp51-d strains, whatever the conditional fork arrest construct. (TIF) [file pgen.1002976.s001.tif]

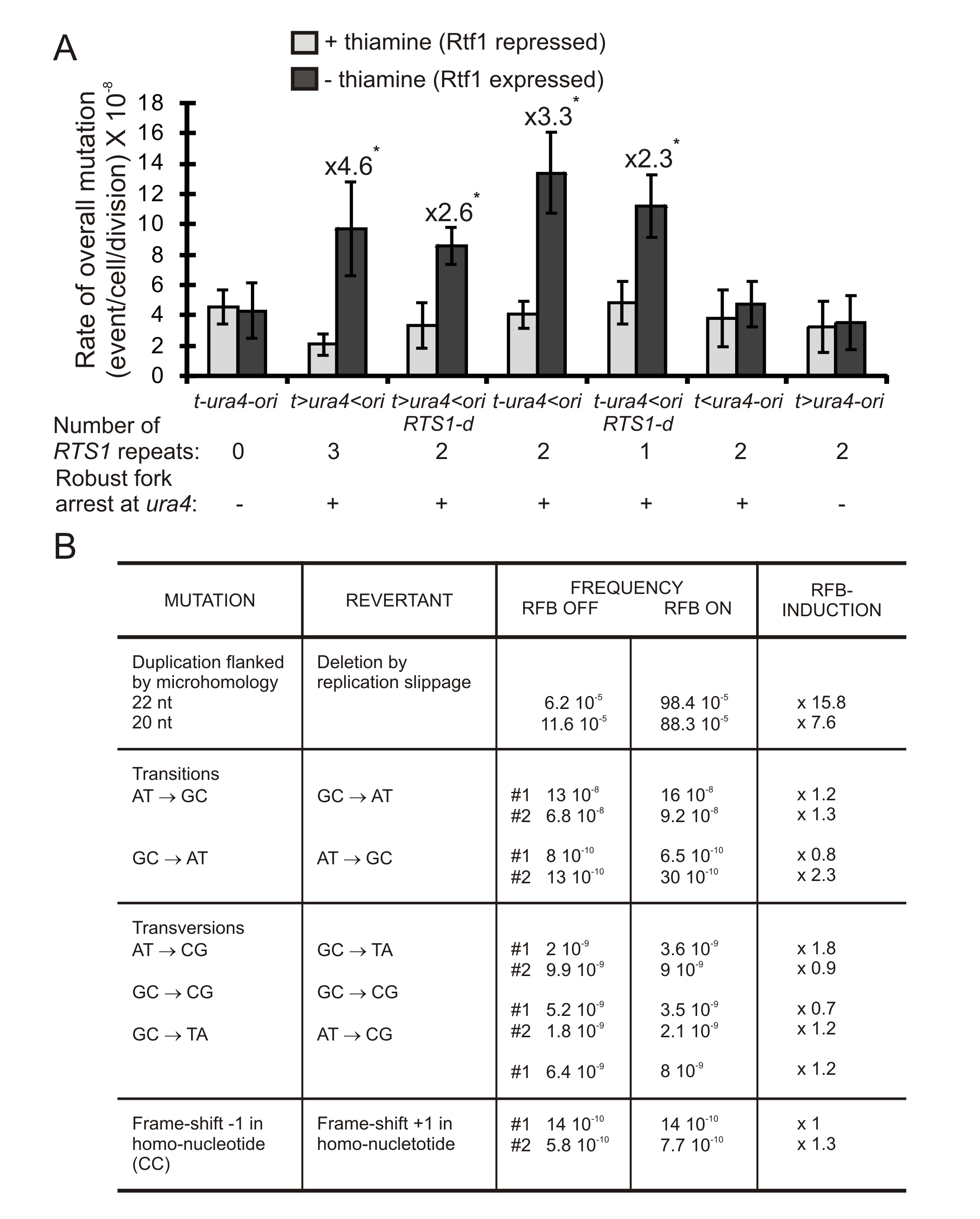

Supplement: Figure S2 — Fork-arrest induces replication slippage. A. The rate of mutation for indicated strains, in the presence (Rtf1 repressed) and in the absence (Rtf1 expressed) of thiamine. Numbers of RTS1 repeats present in the S. pombe genome and the presence of a visible fork arrest (based on 2DGE presented on Figure 1) are given for each strain. The % of mutation events, as determined by the PCR assay and sequencing, was used to balance the rate of ura4 loss. The reported values are means of at least 3 independent median rates. Error bars correspond to SE. Statistically significant fold differences in the rate of mutation events between the Rtf1 “repressed” and “expressed” conditions are indicated with an *. B. The frequency of Ura+ revertants for the indicated strains and conditions. All strains harbour a non-functional ura4 allele due to a single base-substitution or a frame-shift or a duplication of 20 or 22 nt, together with the RTS1-RFB in the t-ura4<ori context. The initial mutations and expected reverted mutations are indicated in the table. #1 and #2 correspond to two independent mutated strains for each type of mutation. (TIF) [file pgen.1002976.s002.tif]

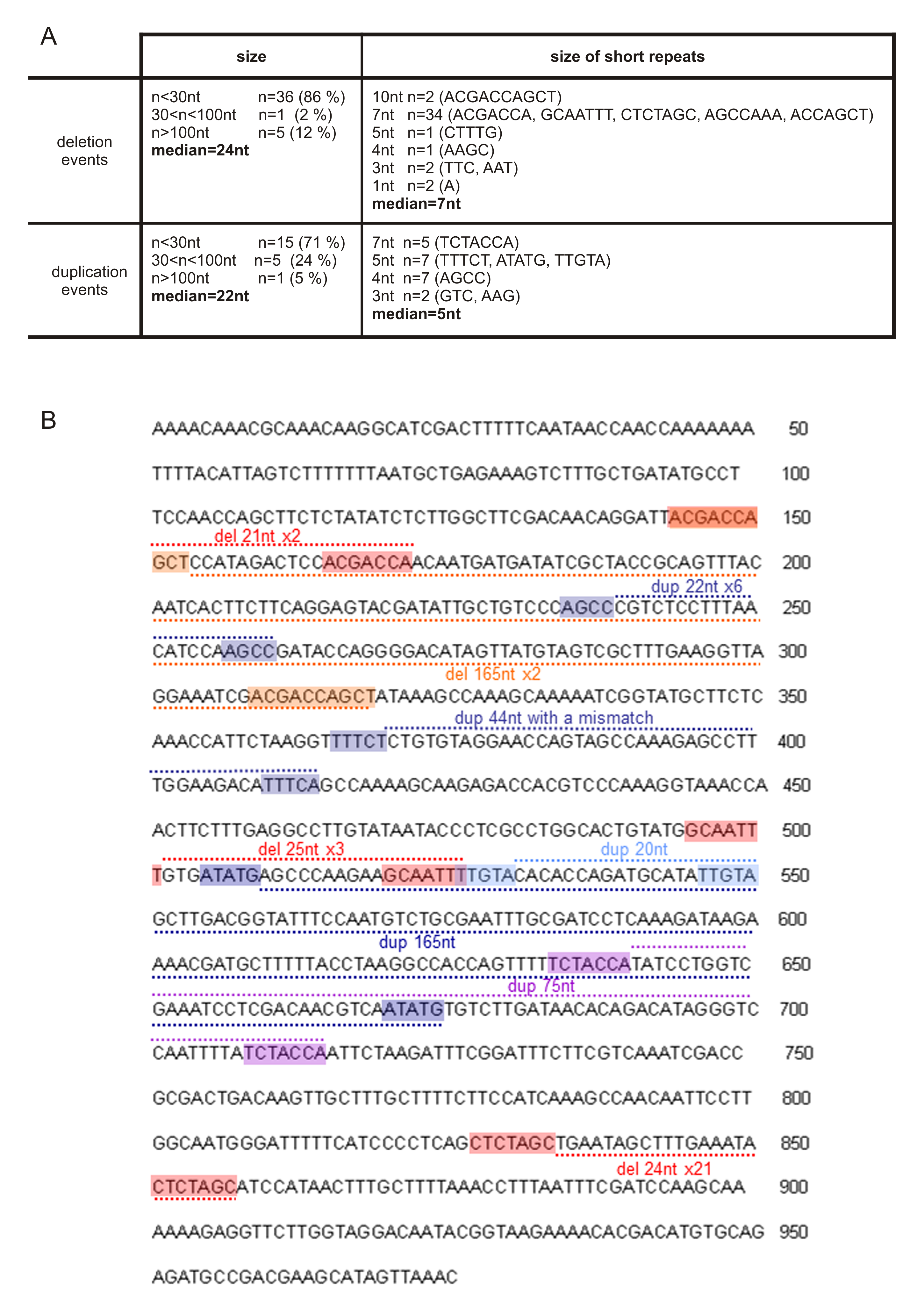

Supplement: Figure S3 — Features of replication slippage induced by fork arrest. A. Table of deletion/duplication and micro-homology features. B. Map of deletion and duplication events observed within the ura4 ORF in the t-ura4<ori construct upon fork arrest. Del and Dup stand for deletion and duplication, respectively. (TIF) [file pgen.1002976.s003.tif]

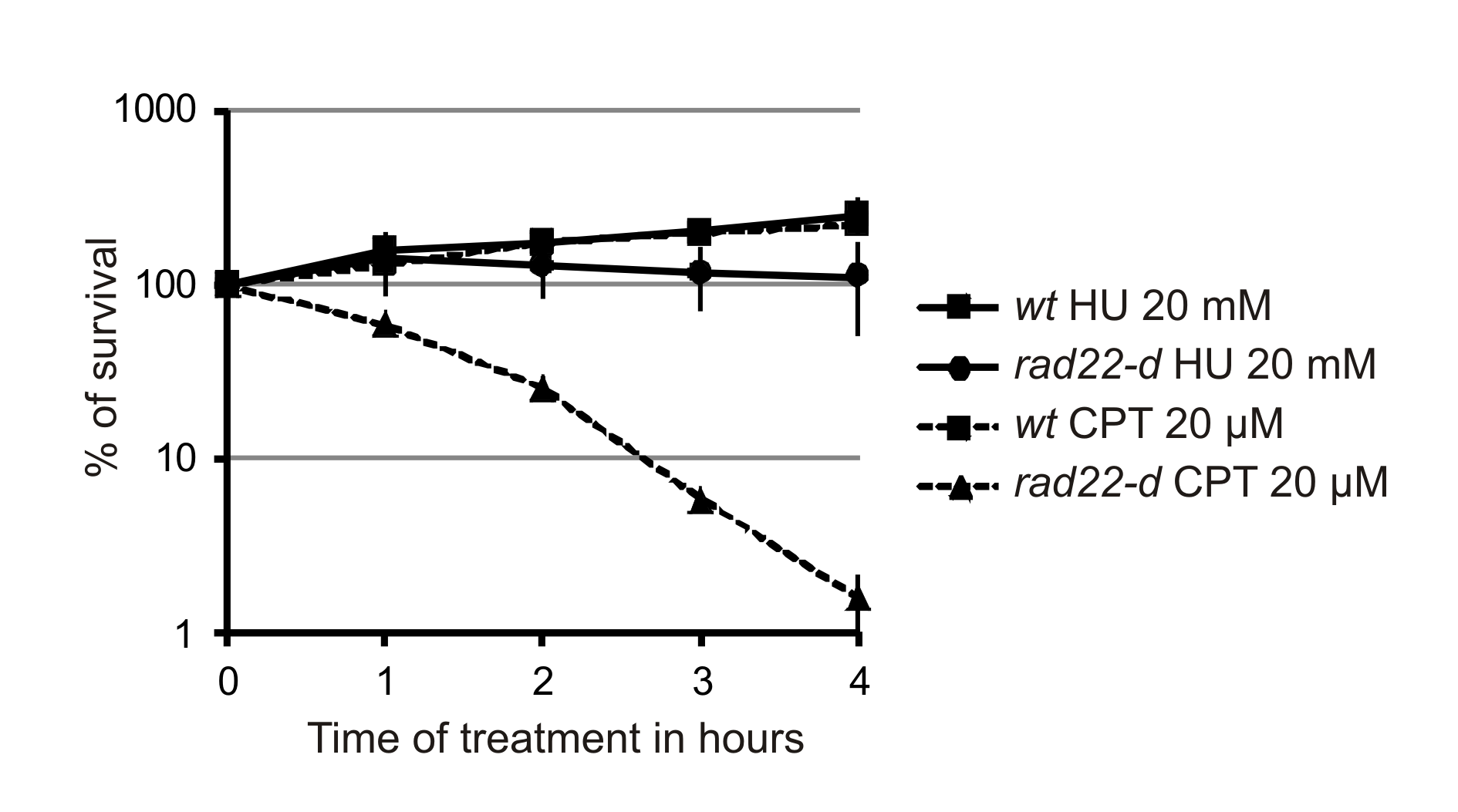

Supplement: Figure S4 — Sensitivity of rad22-d strain to acute exposure to 20 mM of HU or 20 µM of CPT. The values reported are means of two to four independent experiments. Error bars indicate the standard error of the mean (SEM). (TIF) [file pgen.1002976.s004.tif]

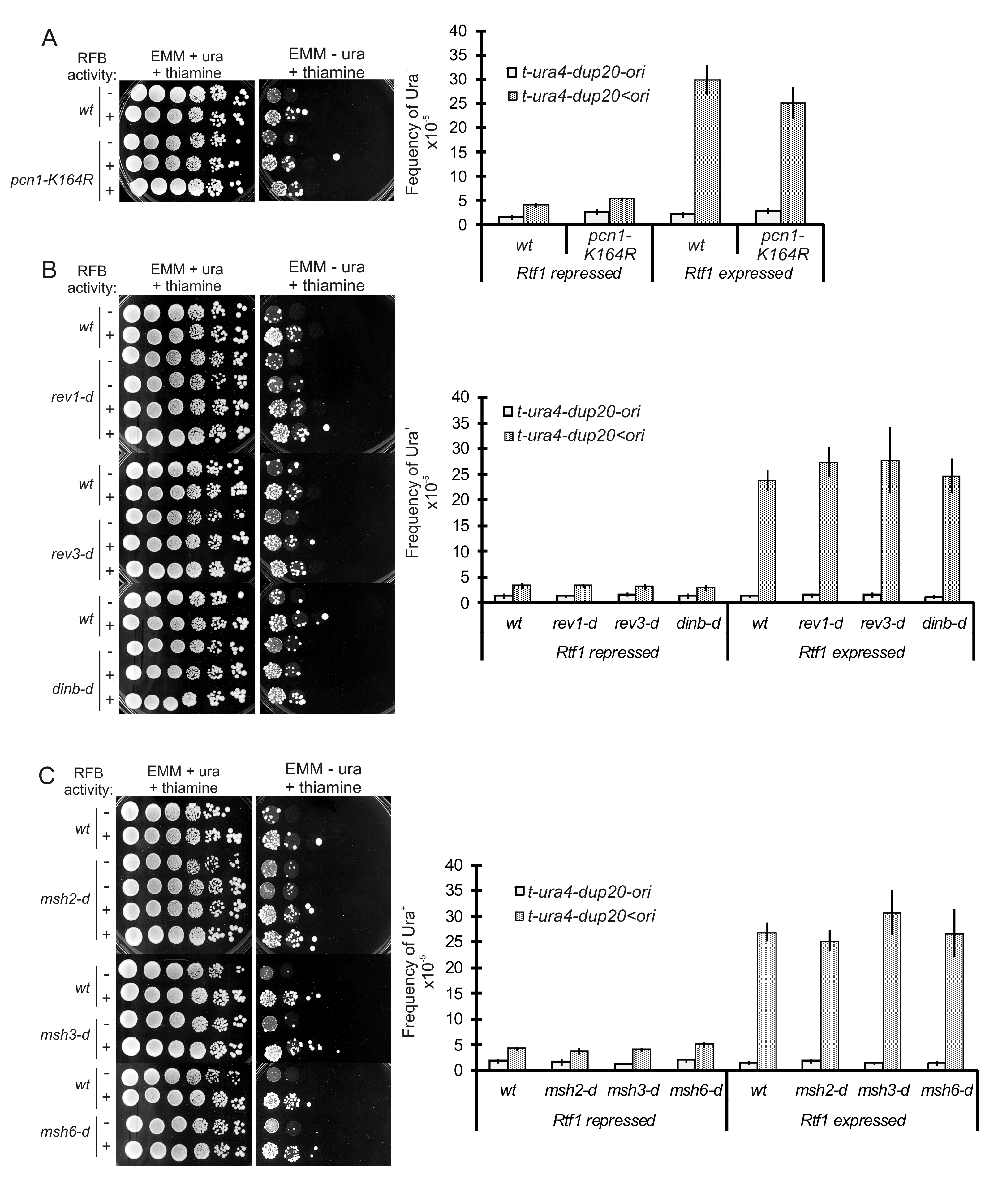

Supplement: Figure S5 — Fork-arrest-induced replication slippage is independent of the post-replication repair and mismatch repair. A–C. Left panels: Serial tenfold-dilutions of indicated strains cultured in thiamine-free medium spotted onto the medium indicated. RTS1-RFB activity “–” refers to the strain t-ura4-dup20-ori and “+” refers to the strain t-ura4-dup20<ori. Right panels: The frequency of Ura+ revertants from the strains indicated (t-ura4-dup20-ori associated or not with the RTS1-RFB) in the conditions indicated. The values reported are means of at least three independent experiments and error bars correspond to SEM. (TIF) [file pgen.1002976.s005.tif]
