# Supplementary material for: Recovery of Arrested Replication Forks by Homologous Recombination Is Error-Prone
Source: PLoS Genet. 2012 Oct 18;8(10):e1002976. doi: 10.1371/journal.pgen.1002976 (PMC3475662; doi:10.1371/journal.pgen.1002976)
Supplement: Table S1 — Strains used in this study. (DOCX) [file pgen.1002976.s006.docx]

Table S1: Strains used in this study.

| Strain | Genotype | Reference |
| --- | --- | --- |
| SL206 | *h^-^ sup35:nmt41: rtf1^+^ ade6-704 leu1-32 ura4^+^* | Lambert *et al.* 2005 |
| SL350 | *h^-^ sup35:nmt41: rtf1^+^ ade6-704 leu1-32 ura4^+^:RTS1 (t-ura4<ori)* | Lambert *et al.* 2005 |
| SL348 | *h^-^ sup35:nmt41: rtf1^+^ ade6-704 leu1-32 RTS1:ura4^+^ (t>ura4-ori)* | Lambert *et al.* 2005 |
| SL229 | *h^-^ sup35:nmt41: rtf1^+^ ade6-704 leu1-32 RTS1:ura4^+^:RTS1 (t>ura4<ori)* | Lambert *et al.* 2005 |
| SL504 | *h^-^ sup35:nmt41: rtf1^+^ ade6-704 leu1-32 oRTS1:ura4^+^ (t<ura4-ori)* | This study |
| SL453 | *h^-^ sup35:nmt41: rtf1^+^ ade6-704 leu1-32 oRTS1:ura4^+^:RTS1o (t<ura4>ori)* | Lambert *et al.* 2010 |
| SL532 | *h^-^ sup35:nmt41: rtf1^+^ ade6-704 leu1-32 RTS1:ura4^+^:RTS1 RTS1::phleoMX6 (t>ura4<ori, RTS1-d)* | This study |
| SL588 | *h^-^ sup35:nmt41: rtf1^+^ ade6-704 leu1-32 oRTS1:ura4^+^:RTS1o RTS1::phleoMX6 (t<ura4>ori, RTS1-d)* | This study |
| SL587 | *h^-^ sup35:nmt41: rtf1^+^ ade6-704 leu1-32 ura4^+^:RTS1 RTS1::phleoMX6 (t-ura4<ori, RTS1-d)* | This study |
| SL558 | *h^-^ sup35:nmt41: rtf1^+^ ade6-704 leu1-32 RTS1:ura4^+^:RTS1 rqh1::kanMX6 (t>ura4<ori)* | Lambert *et al.* 2010 |
| SL625 | *h^-^ sup35:nmt41: rtf1^+^ ade6-704 leu1-32 ura4^+^:RTS1 rqh1::kanMX6 (t-ura4<ori)* | Lambert *et al.* 2010 |
| SL564 | *h^-^ sup35:nmt41: rtf1^+^ ade6-704 leu1-32 RTS1:ura4^+^:RTS1 rad22::natMX6 (t>ura4<ori)* | Lambert *et al.* 2005 |
| SL677 | *h^-^ sup35:nmt41: rtf1^+^ ade6-704 leu1-32 ura4^+^:RTS1 rad22::natMX6 (t-ura4<ori)* | Lambert *et al.* 2005 |
| SL534 | *h^-^ sup35:nmt41: rtf1^+^ ade6-704 leu1-32 RTS1:ura4^+^:RTS1 rhp51::kanMX6 (t>ura4<ori)* | Lambert *et al.* 2005 |
| SL398 | *h^-^ sup35:nmt41: rtf1^+^ ade6-704 leu1-32 ura4^+^:RTS1 rhp51::kanMX6 (t-ura4<ori)* | Lambert *et al.* 2005 |
| SL938 | *h^-^ sup35:nmt41: rtf1^+^ ade6-704 leu1-32 ura4-603dup22:RTS1 (t-ura4-dup22<ori)* | This study |
| SL 939 | *h^-^ sup35:nmt41: rtf1^+^ ade6-704 leu1-32 ura4-313dup20:RTS1 (t-ura4-dup20<ori)* | This study |
| YC94 | *h^-^ sup35:nmt41: rtf1^+^ ade6-704 leu1-32 ura4-T443C:RTS1 (t-ura4-T443C<ori)* | This study |
| YC95 | *h^-^ sup35:nmt41: rtf1^+^ ade6-704 leu1-32 ura4-T443C:RTS1 (t-ura4-T443C<ori)* | This study |
| YC96 | *h^-^ sup35:nmt41: rtf1^+^ ade6-704 leu1-32 ura4-C795T:RTS1 (t-ura4-C795T<ori)* | This study |
| YC97 | *h^-^ sup35:nmt41: rtf1^+^ ade6-704 leu1-32 ura4-C19T:RTS1 (t-ura4-C19T<ori)* | This study |
| YC100 | *h^-^ sup35:nmt41: rtf1^+^ ade6-704 leu1-32 ura4-T437G:RTS1 (t-ura4-T437G<ori)* | This study |
| YC101 | *h^-^ sup35:nmt41: rtf1^+^ ade6-704 leu1-32 ura4-T143G:RTS1 (t-ura4-T143G<ori)* | This study |
| YC102 | *h^-^ sup35:nmt41: rtf1^+^ ade6-704 leu1-32 ura4-G664C:RTS1 (t-ura4-G664C<ori)* | This study |
| YC103 | *h^-^ sup35:nmt41: rtf1^+^ ade6-704 leu1-32 ura4-G35C:RTS1 (t-ura4-G35C<ori)* | This study |
| YC104 | *h^-^ sup35:nmt41: rtf1^+^ ade6-704 leu1-32 ura4-1G at 626:RTS1 (uraR)* | This study |
| YC105 | *h^-^ sup35:nmt41: rtf1^+^ ade6-704 leu1-32 ura4-1G at 427:RTS1 (t-ura4-+1G at 427<ori)* | This study |
| YC108 | *h^-^ sup35:nmt41: rtf1^+^ ade6-704 leu1-32 ura4-C135A:RTS1 (t-ura4-C135A<ori)* | This study |
| YC5 | *h^-^ sup35:nmt41: rtf1^+^ ade6-704 leu1-32 ura4-313dup20 (t-ura4-dup20-ori)* | This study |
| YC13 | *h^-^ sup35:nmt41: rtf1^+^ ade6-704 leu1-32 ura4-313dup20:RTS1 (t-ura4-dup20<ori)* | This study |
| YC21 | *h^-^ sup35:nmt41: rtf1^+^ ade6-704 leu1-32 oRTS1:ura4-313dup20 (t<ura4-dup20-ori)* | This study |
| YC41 | *h^-^ sup35:nmt41: rtf1^+^ ade6-704 leu1-32 ura4-603dup22:RTS1 (t-ura4-dup22<ori)* | This study |
| YC50 | *h^-^ sup35:nmt41: rtf1^+^ ade6-704 leu1-32 oRTS1:ura4-603dup22 (t<ura4-dup22-ori)* | This study |
| YC65 | *h^-^smt0 sup35:nmt41: rtf1^+^ ade6-704 leu1-32 ura4-313dup20 rad50::kanMX6 (t-ura4-dup20-ori)* | This study |
| YC67 | *h^-^smt0 sup35:nmt41: rtf1^+^ ade6-704 leu1-32 ura4-313dup20:RTS1 rad50::kanMX6 (t-ura4-dup20<ori)* | This study |
| YC76 | *h^-^smt0 sup35:nmt41: rtf1^+^ ade6-704 leu1-32 ura4-313dup20 rhp51::kanMX6 (t-ura4-dup20-ori)* | This study |
| YC80 | *h^-^smt0 sup35:nmt41: rtf1^+^ ade6-704 leu1-32 ura4-313dup20:RTS1 rhp51::kanMX6 (t-ura4-dup20<ori)* | This study |
| YC86 | *h^-^smt0 sup35:nmt41: rtf1^+^ ade6-704 leu1-32 ura4-313dup20 rad22::natMX6 (t-ura4-dup20-ori)* | This study |
| YC90 | *h^-^smt0 sup35:nmt41: rtf1^+^ ade6-704 leu1-32 ura4-313dup20:RTS1 rad22::natMX6 (t-ura4-dup20<ori)* | This study |
| YC111 | *h^-^smt0 sup35:nmt41: rtf1^+^ ade6-704 leu1-32 ura4-313dup20 rad22::natMX6 rhp51::kanMX6 (t-ura4-dup20-ori)* | This study |
| YC115 | *h^-^smt0 sup35:nmt41: rtf1^+^ ade6-704 leu1-32 ura4-313dup20:RTS1 rad22::natMX6 rhp51::kanMX6 (t-ura4-dup20<ori)* | This study |
| YC163 | *h^-^smt0 sup35:nmt41: rtf1^+^ ade6-704 leu1-32 ura4-313dup20 pcn1-K164R (t-ura4-dup20-ori)* | This study |
| YC166 | *h^-^smt0 sup35:nmt41: rtf1^+^ ade6-704 leu1-32 ura4-313dup20:RTS1 pcn1-K164R (t-ura4-dup20<ori)* | This study |
| YC143 | *h^-^smt0 sup35:nmt41: rtf1^+^ ade6-704 leu1-32 ura4-313dup20 rev1::kanMX6 (t-ura4-dup20-ori)* | This study |
| YC139 | *h^-^smt0 sup35:nmt41: rtf1^+^ ade6-704 leu1-32 ura4-313dup20:RTS1 rev1::kanMX6 (t-ura4-dup20<ori)* | This study |
| YC146 | *h^-^smt0 sup35:nmt41: rtf1^+^ ade6-704 leu1-32 ura4-313dup20 rev3::hygMX6 (t-ura4-dup20-ori)* | This study |
| YC150 | *h^-^smt0 sup35:nmt41: rtf1^+^ ade6-704 leu1-32 ura4-313dup20:RTS1 rev3::hygMX6 (t-ura4-dup20<ori)* | This study |
| YC153 | *h^-^smt0 sup35:nmt41: rtf1^+^ ade6-704 leu1-32 ura4-313dup20 dinb::hygMX6 (t-ura4-dup20-ori)* | This study |
| YC157 | *h^-^smt0 sup35:nmt41: rtf1^+^ ade6-704 leu1-32 ura4-313dup20:RTS1 dinB::hygMX6 (t-ura4-dup20<ori)* | This study |
| YC158 | *h^+^ sup35:nmt41: rtf1^+^ ade6-704 leu1-32 ura4-313dup20 msh2::kanMX6 (t-ura4-dup20-ori)* | This study |
| YC161 | *h^+^ sup35:nmt41: rtf1^+^ ade6-704 leu1-32 ura4-313dup20:RTS1 msh2::kanMX6 (t-ura4-dup20<ori)* | This study |
| YC117 | *h^+^ sup35:nmt41: rtf1^+^ ade6-704 leu1-32 ura4-313dup20 msh3::hygMX6 (t-ura4-dup20-ori)* | This study |
| YC121 | *h^-^smt0 sup35:nmt41: rtf1^+^ ade6-704 leu1-32 ura4-313dup20:RTS1 msh3::hygMX6 (t-ura4-dup20<ori)* | This study |
| YC125 | *h^-^smt0 sup35:nmt41: rtf1^+^ ade6-704 leu1-32 ura4-313dup20 msh6::natMX6 (t-ura4-dup20-ori)* | This study |
| YC127 | *h^-^smt0 sup35:nmt41: rtf1^+^ ade6-704 leu1-32 ura4-313dup20:RTS1 msh6::natMX6 (t-ura4-dup20<ori)* | This study |
